# FOXO3 Is a Glucocorticoid Receptor Target and Regulates LKB1 and Its Own Expression Based on Cellular AMP Levels via a Positive Autoregulatory Loop

**DOI:** 10.1371/journal.pone.0042166

**Published:** 2012-07-27

**Authors:** Nicolas Lützner, Hubert Kalbacher, Anja Krones-Herzig, Frank Rösl

**Affiliations:** 1 Research Program Infections and Cancer, German Cancer Research Center (DKFZ), Heidelberg, Germany; 2 Interfaculty Institute for Biochemistry, University of Tübingen, Tübingen, Germany; 3 Research Program Cell Biology and Tumor Biology, German Cancer Research Center (DKFZ), Heidelberg, Germany; North Carolina State University, United States of America

## Abstract

FOXO3 is a transcription factor involved in the regulation of multiple physiological processes including cell cycle arrest, apoptosis, oxidative stress-response and energy metabolism. Although much is known about its post-translational modification, the transcriptional regulation of FOXO3, as well as the cross-talk between transcription and post-translational events, is still poorly understood. In the present study, we show that FOXO3 is an immediate early glucocorticoid receptor (GR) target, whose transcription is even further enhanced by conditions that mimic metabolic stress. Induction of FOXO3 transcription by GR-binding steroids was reversed by concomitant treatment with the GR antagonist RU-486, but further enhanced by stimuli that activate the AMP-activated protein kinase (AMPK). Analysis of genomic DNA and chromatin immunoprecipitation, as well as luciferase reporter assays, revealed two functional glucocorticoid responsive elements within the FOXO3 promoter. Furthermore, we provide functional evidence for a phosphorylation switch that explains how glucocorticoids induce transcriptional activation of the gene but subsequently inactivate the corresponding protein by site-specific phosphorylation. Only when AMPK is stimulated, pre-existing FOXO3 becomes reverted toward an active form. Energy deprived conditions thus activate FOXO3 on two different levels, namely transcriptional and post-translational. In that way, FOXO3 acts as a metabolic stress sensor that coordinates expression of LKB1, the master upstream kinase involved in metabolic sensing, depending on the energy status of the cell. Additionally, we show that FOXO3 binds and activates its own promoter via a positive autoregulatory feedback loop. In conclusion, our data explain how catabolic glucocorticoid hormones and high intracellular AMP levels cooperate in inducing FOXO3 transcription and in activating the corresponding protein.

## Introduction

Forkhead box transcription factors constitute a family of evolutionary well conserved proteins that share a common DNA-binding domain, the so-called forkhead box [1]–[3]. FOXO3 belongs to subfamily O of forkhead box transcription factors (FOXO) [4], whose members induce cell cycle arrest [5], [6], DNA damage repair [7], [8] and apoptosis [9], [10]. Consistent with their ability to block cell growth, inactivation of FOXO proteins is thought to be a critical event in oncogenic transformation [11], [12]. Deletion of all FOXO1, FOXO3, and FOXO4 alleles in adult mice induced a cancer prone phenotype, supporting a tumour suppressing function of these proteins [13]. The identification of chromosomal breakpoints within FOXO genes, producing hybrid proteins in human tumours further supports this idea [14]–[16]. In addition to their role in cell cycle regulation, FOXO transcription factors also regulate glucose metabolism in various organs [17]–[19] and increase the resistance to oxidative stress [20]–[22]. Furthermore, the FOXO orthologue DAF-16 controls life span extension in Caenorhabditis elegans together with homologues of the human Insulin receptor and the phosphatidylinositol 3-kinase (DAF-2 and AGE-1) [23], [24].

Similar to the pathway in C. elegans, human FOXO3 also becomes inactivated by insulin and other related growth factors through direct phosphorylation by the protein kinase B (PKB, also named c-Akt) [25], a serine/threonine kinase that is activated by the phosphatidylinositol 3-kinase (PI3-kinase) [26], [27]. In addition to PKB, the serum and glucocorticoid-inducible kinase 1 (SGK-1), another downstream effector of the PI3-kinase [28], also inactivates FOXO3 by direct phosphorylation of the same three amino acids (Thr 32, Ser 253 and Ser 315) [29]. SGK-1 was originally identified as an immediate early response gene induced following serum or glucocorticoid treatment [30]. Furthermore, it was shown by expression of a SGK-1 short interfering RNA that glucocorticoid-mediated induction of SGK-1 is sufficient to inactivate FOXO3 even in the absence of serum [31]. In the absence of both, serum and glucocorticoids, FOXO3 reside in the nucleus [25] and activates target genes that either promote apoptosis (e.g. Fas ligand, Bim, and TRAIL) [9], [10], [25] or inhibit cell cycle progression (e.g. p27 KIP1 and p21 WAF1) [5], [32]. Recently, we identified a functional connection between FOXO3 and the transcriptional activation of the tumour suppressor gene Liver Kinase B1 (LKB1) [33], which is known to block cell cycle progression [34] and which is frequently lost in many forms of human tumours, including cervical cancer [35], [36].

Mechanisms that explain how LKB1 inhibits cell cycle and tumour progression could be mainly attributed to direct phosphorylation of different AMP-activated protein kinase (AMPK) family members [37]–[40]. AMPK is allosterically activated by high AMP concentrations [41], [42], facilitating phosphorylation of the activating residue Thr 172 by LKB1 [43]. Once activated, AMPK acts as a metabolic stress-sensor that switches on ATP-producing pathways and simultaneously inhibits ATP-consuming processes in order to sustain energy homeostasis [43]. This is mainly achieved by rapid phosphorylation of metabolic target enzymes, such as acetyl-CoA carboxylase (ACC) [41], but also by modulation of transcriptional regulators. In this context, it should be noted that AMPK phosphorylation of FOXO3 has been shown to activate its transcriptional activity [44]. When intracellular energy levels are restored AMPK is allosterically inactivated by ATP [41], [42].

The intracellular ATP/ADP ratio and the regulation of anabolic and catabolic pathways in response to changing environmental conditions are not only controlled by AMPK, but also by metabolic hormones. The anabolic hormone Insulin, for instance, signals the liver to increase glucose uptake, glycogen synthesis, and to attenuate gluconeogenesis [45]. Conversely, glucagon and glucocorticoids signal the liver to up-regulate gluconeogenesis [45]–[49] in order to restore cellular energy levels by providing glucose to the periphery [50]. As described above, insulin control of metabolism is largely mediated through PKB [23], [27], which phosphorylates and inactivates FOXO transcription factors [25]. Beside this well characterized post-translational control by insulin, several reports also describe a regulation of FOXOs by glucocorticoids [51]–[55]. Glucocorticoid-induced muscle atrophy is mediated by FOXO transcription factors [51], [52] mostly due to inhibition of PKB [51]–[53] and recent findings even suggest that FOXO1 and FOXO3 expression is up-regulated upon glucocorticoid treatment [55]. However, transcriptional regulation of FOXO3 is still poorly understood and it still remains to be elucidated whether FOXO3 is a direct GR target gene.

Here we show that glucocorticoids and high intracellular AMP levels directly activate FOXO3 and act in a completely opposite way than insulin. We demonstrate that FOXO3 is an immediate early glucocorticoid receptor target gene that coordinates expression of LKB1 and other FOXO3 target genes depending on the metabolic status of the cell. Furthermore, we provide evidence that glucocorticoid-induced transcriptional activation of FOXO3 is further enhanced by AMPK activating stimuli and that FOXO3 binds and activates its own promoter via a positive autoregulatory feedback loop. In conclusion, our data suggests that conditions of metabolic stress, characterized by high AMP and glucocorticoid concentrations, activate FOXO3 and thereby act antagonistically to insulin and related growth factors.

## Results

### SGK-1 Negatively Regulates LKB1 Expression via FOXO3 Transcription Factor

**Figure 1:**
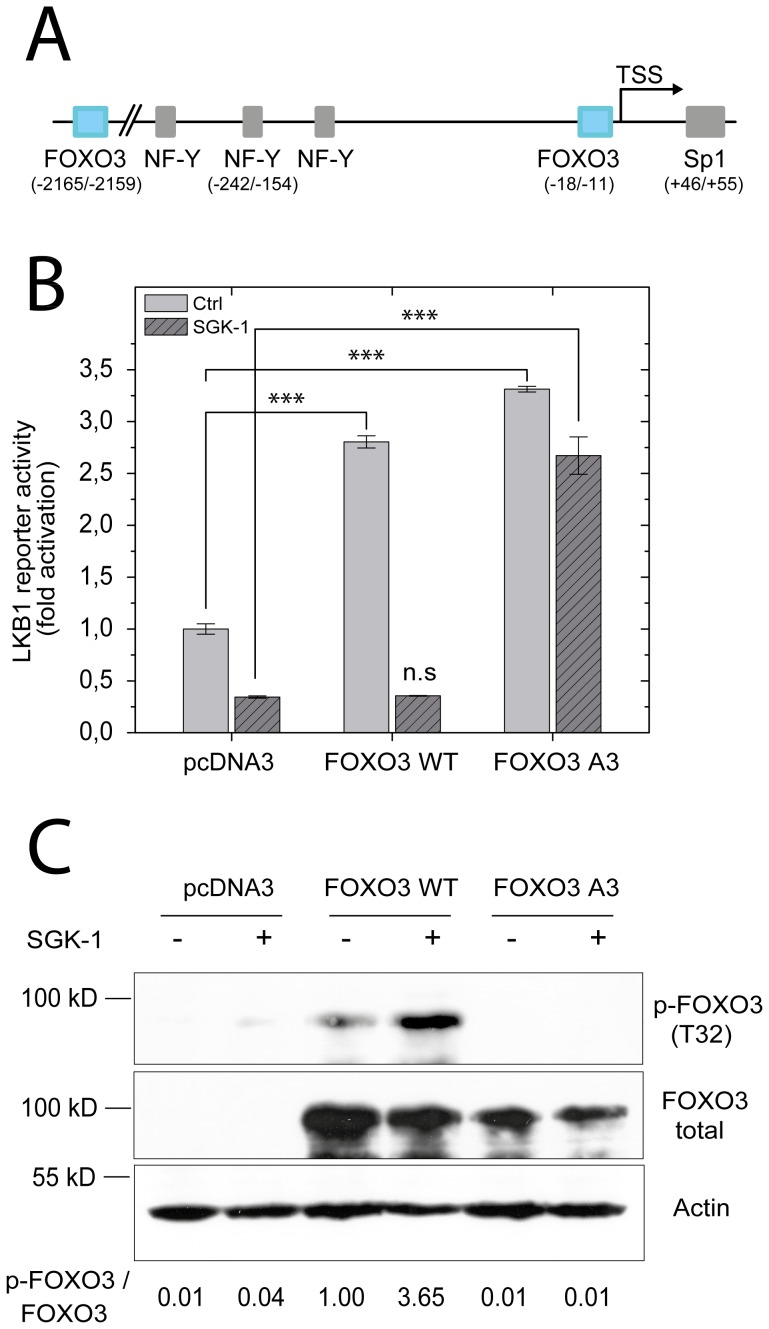
SGK-1 inhibits FOXO3 transcriptional activity and subsequently LKB1 promoter activation. (**A**) Illustration of the LKB1 promoter region, extending from nucleotide position -2537 to +727 relative to the transcription start site. Positions of the transcription start site (TSS) and binding sites of transcription factors involved in LKB1 regulation (FOXO3, NF-Y and Sp1) as well as their positions relative to the transcription start site are indicated in brackets. (**B**) LKB1 reporter activity in HEK293 cells after ectopic expression of SGK-1 (dark grey) is compared to the activity obtained from co-transfections with the empty vector (Ctrl, light grey). Reporter activity is expressed as fold of luciferase activity (relative light units normalized to renilla luciferase activity) obtained from co-transfection of the plasmid containing the LKB1 promoter (nucleotides −2537 to +727) together with the empty expression vector (pcDNA3). Instead of the empty vector the same amount of either a FOXO3 wild-type expression plasmid (FOXO3 WT) or a SGK-1 phosphorylation site deficient triple mutant (T32A/S253A/S315A) of FOXO3 (FOXO3 A3) has been co-transfected. Each bar represents the means ± standard deviation of three measurements. (**C**) Western blot analysis of the same lysates used in the LKB1 reporter assay described in (B) using antibodies against phospho-FOXO3 (T32), total FOXO3 and actin. The ratios of p-FOXO3/FOXO3 for each experimental condition are indicated. The shown blot is a representative of three independent experiments.

Performing a functional analysis of the human LKB1 promoter, several important DNA-binding proteins that are critical for LKB1 gene expression were identified [33] (Figure 1A). Beside NF-Y and Sp1, representing two of the most prominent transcription factors involved in the regulation of a large number of genes [56], [57], we also showed that FOXO3 acts as key regulatory factor in LKB1 transcription. Furthermore, we demonstrated that a SGK-1 phosphorylation site deficient mutant of FOXO3 (FOXO3 A3: triple mutant T32A/S253A/S315A) was more potent in inducing LKB1 promoter activity than the wild type form, suggesting that SGK-1 might be a negative regulator of LKB1 expression [33].

To prove this assumption, HEK 293 cells were transfected with a LKB1 promoter-driven luciferase reporter together with a SGK-1 expression vector and a plasmid encoding either wild-type FOXO3 (FOXO3 WT) or a mutant form of FOXO3 that cannot be phosphorylated (FOXO3 A3) by SGK-1. 24 hours after transfection luciferase activity was measured (Figure 1B) and FOXO3 protein levels were analysed by western blotting (Figure 1C). As shown in panel B, LKB1 promoter activity was increased approximately 3 to 3.5 fold after FOXO3 or FOXO3 A3 overexpression. However, induction was changed after co-transfection of the SGK-1 expression plasmid. While the transcriptional activity of the endogenous FOXO3 and transfected FOXO3 WT was decreased after SGK-1 expression, luciferase activity of FOXO3 A3 expressing cells was not affected. As shown in Figure 1C, SGK-1 mediated inhibition of the FOXO3 WT driven LKB1 reporter induction was accompanied by an increased phosphorylation of the threonine 32 residue (T32) within the FOXO3 WT protein. These observations suggest that SGK-1 induced FOXO3 phosphorylation inhibits its transcriptional activity and thereby decreases LKB1 promoter activity.

### Glucocorticoid-induced Activation of SGK-1 Inhibits the Transcription of LKB1 and Other FOXO3 Target Genes

**Figure 2:**
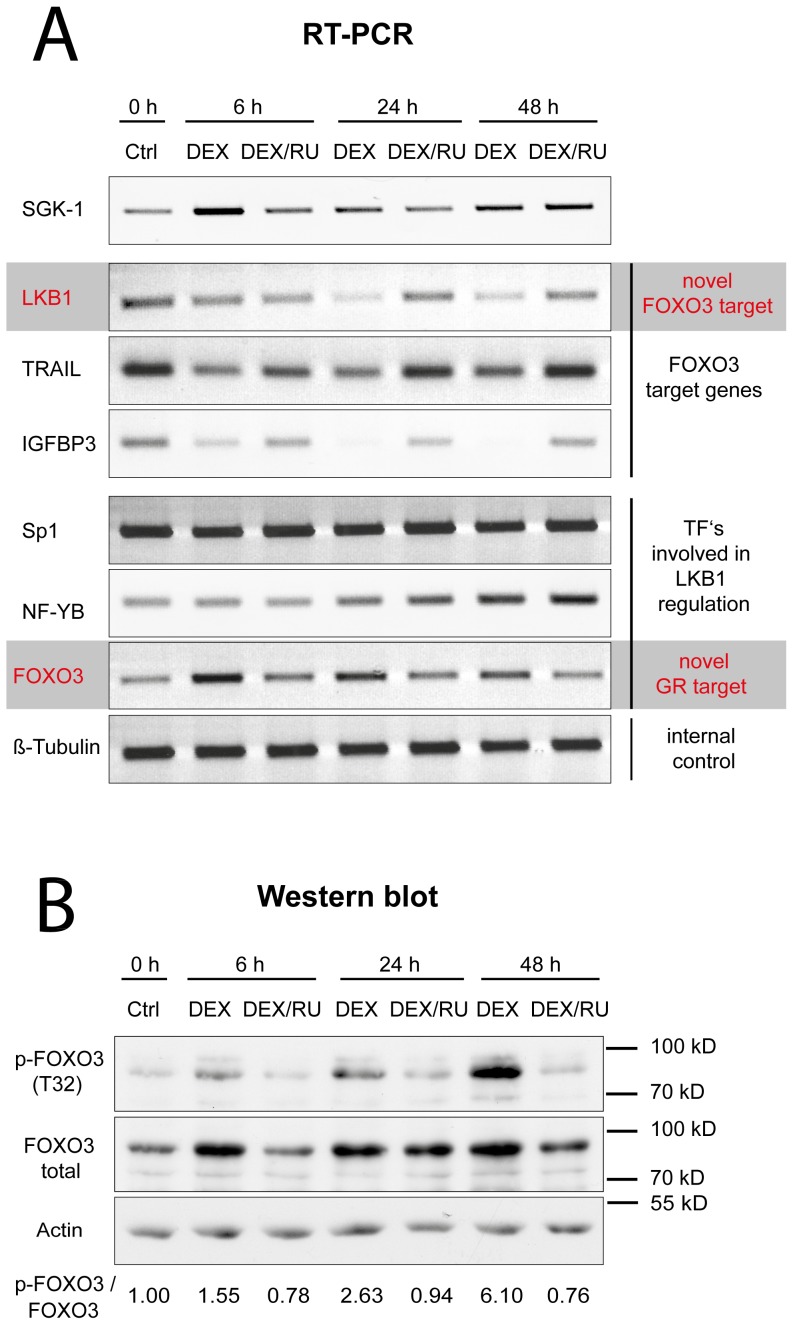
Glucocorticoid-mediated SGK-1 induction inhibits LKB1 transcription through FOXO3 (T32) phosphorylation. MCF-10A cells were treated with either vehicle (ethanol) (Ctrl), 1 µM dexamethasone (DEX) or a combination of 1 µM dexamethasone and 1 µM of the GR antagonist RU-486 (DEX/RU) for 0, 6, 24, and 48 h. (**A**) Relative mRNA levels of SGK-1, FOXO3 target genes (LKB1, TRAIL, IGFBP3) and the transcription factors involved in LKB1 regulation (Sp1, NF-YB, FOXO3). β-tubulin was used as internal control. RT-PCR products were analysed in a 1% agarose gel after ethidium bromide staining. (**B**) Relative protein levels of phospho-FOXO3 (T32), total FOXO3 and actin as internal control were analysed by western blotting. The ratios of p-FOXO3/FOXO3 for each experimental condition are indicated.

Since SGK-1 is an immediate early response glucocorticoid receptor (GR) target gene [30] and GR-mediated SGK-1 induction can also inhibit FOXO3 activity by phosphorylation at T32 [31], we next analysed the impact of GR activation on LKB1 expression. For this purpose, glucocorticoid-responsive MCF-10A mammary epithelial cells [58] were incubated with dexamethasone (Dex) for different periods of time. Under those conditions, SGK-1 was transiently stimulated after 6 hours and remained elevated in quantities still above the initial steady-state level detected prior to glucocorticoid treatment (Figure 2A). SGK-1 induction was accompanied by a temporary delayed inhibitory FOXO3 phosphorylation at T32 (Figure 2B). While after 6 hours only a slight FOXO3 T32 phosphorylation could be discerned, apparently just correlating with an increase of total protein level *per se*, FOXO3 became strongly phosphorylated between 24 and 48 hours (Figure 2B).

Consistent with the fact that phosphorylation of FOXO3 at T32 results in nuclear exclusion of the protein [25], [31], there was not only an inhibitory effect on known FOXO3 target genes such as the Insulin like growth factor binding protein 3 (IGFBP-3) and the TNF-related apoptosis-inducing ligand (TRAIL) [10], [31], but also on LKB1 expression (Figure 2A). Conversely, GR-mediated repression of FOXO3 target genes, as well as the induction of SGK-1 and FOXO3 T32 phosphorylation, was inhibited by the progesterone receptor (PR) and GR antagonist RU-486 [59]. Although RU-486 inactivates also the PR, it can yet be considered as GR specific under these experimental conditions, since MCF-10A cells do not express PR-A or PR-B [60].

To exclude whether down-regulation of LKB1 expression was merely caused by simultaneous repression of transcription factors involved in LKB1 regulation [33], the transcription of Sp1, NF-YB and FOXO3 was monitored by RT-PCR. While Sp1 and NF-YB mRNA levels were not affected, FOXO3 transcription, like SGK-1 was induced after Dex treatment (Figure 2A). This induction correlates with the elevated FOXO3 protein levels (Figure 2B) and could be reversed by concomitant RU-486 treatment. In conclusion, these results indicate that post-translational FOXO3 modification directly affects LKB1 expression upon GR activation and suggests that FOXO3 might be a direct target gene of GR activation.

### FOXO3 is an Immediate Early Glucocorticoid Receptor Target Gene

**Figure 3:**
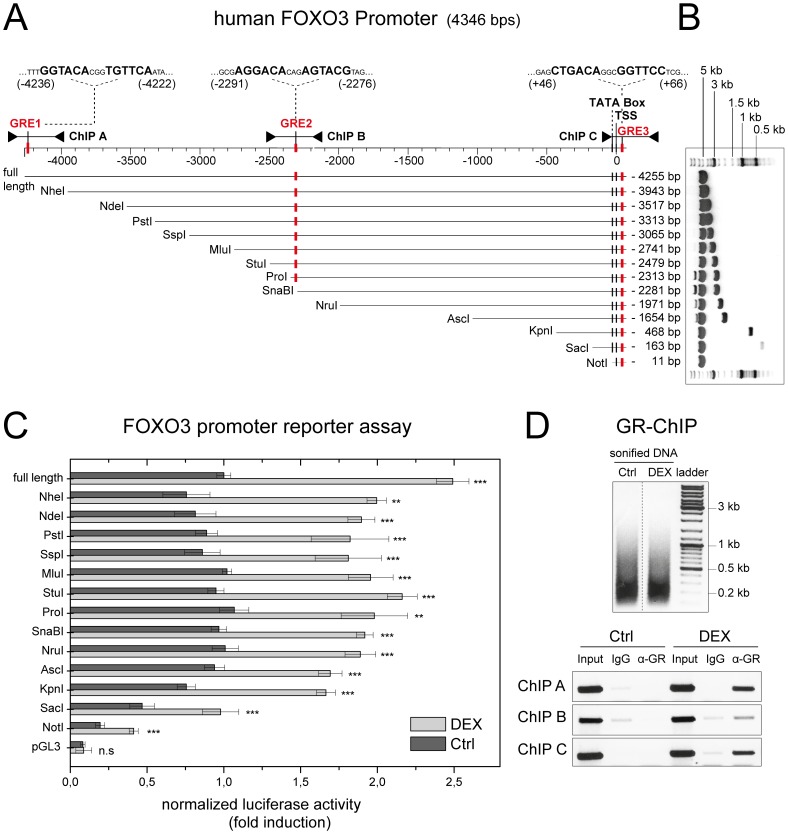
Glucocorticoid receptor directly binds and activates the FOXO3 promoter. (A) Illustration of the FOXO3 promoter region, extending from nucleotide position −4255 to +91 relative to the transcription start site, matched with deletion constructs generated by the indicated restriction enzymes. The 5′-extension relative to the transcription start site of each construct in base pairs (bp) is given on the right side (full length: −4255 to NotI: −11 respectively). The 3′-end at +91 is identical for each construct. The position of the TATA box, the transcription start site (TSS) and the potential glucocorticoid response elements (GRE 1, 2, 3) are indicated. The PCR products amplified in the ChIP assay (ChIP A, B, C) as well as the sequence of the potential GREs are marked above the illustration. (B) Not I restriction digestion of all constructed FOXO3 promoter deletion constructs separated in a 1% agarose gel. (C) FOXO3 promoter reporter assay in A549 cells treated with either vehicle (Ctrl, dark grey) or 1 µM dexamethasone (DEX, light grey) for 12 h. Luciferase activity of all FOXO3 promoter deletion constructs (relative light units normalized against renilla luciferase activity) is expressed as fold of the signal obtained with the plasmid containing the full length promoter (−4255 to +91) without dexamethasone treatment. Assays were performed in triplicates. The error bars denote mean ± standard deviation. (D) ChIP assay in MCF-10A cells treated with either 1 µM dexamethasone (DEX) or vehicle (Ctrl) for 3 h. DNA was sonicated to an average size of <500 bp and run on a 1% (wt/vol) agarose gel (upper panel). PCR products specific for the different potential GREs within the FOXO3 promoter (ChIP A, B, C) were amplified from sonicated DNA of 1/10 of the starting material (Input, positive control) as well as from sonicated DNA after ChIP with either a non-specific antibody (IgG, negative control) or with an antibody against the glucocorticoid receptor (α-GR). Products were visualized within the linear range of the reaction by ethidium bromide staining on a 1% (wt/vol) agarose gel (lower panel). The figure shows a representative of three independent experiments.

The data presented above indicated that glucocorticoids regulate FOXO3 not only post-translationally [29], [31] but also on transcriptional level (Figure 2A). To investigate whether increased FOXO3 transcription upon Dex addition was mediated by a direct binding of GR to the FOXO3 promoter, the genomic sequence upstream of the human FOXO3 gene was monitored for the presence of potential glucocorticoid response elements (GREs). Bioinformatic analysis revealed three potential GRE sequences (GRE1∶5′-GGTACAcggTGTTCA-3′; GRE2∶5′-AGGACAcagAGTACG-3′; GRE3∶5′-CTGACAggcGGTTCC-3′) with close homology to the consensus GRE (5′-GGTACAnnnTGTTCT-3′) [61]. These elements were located at approximately −4 kb, −2 kb and +50 bp relative to the transcription start site (see schematic overview, Figure 3A).

To confirm functionality of the GREs, the human FOXO3 promoter ranging from −4255 bp to +89 bp was amplified from a bacterial artificial chromosome by PCR and cloned in front of a luciferase reporter gene. Moreover, multiple deletion constructs were generated as indicated by the respective restriction enzymes (Figure 3A and B). These reporter plasmids (referred according to their 5′-end as defined by the enzyme used for deletion) or the corresponding promoterless control construct (referred as “pGL3”) were then transfected and luciferase activity was measured following incubation with or without Dex for 12 h (Figure 3C). Due to the low transfection efficiency of MCF-10A cells to all available methods, glucocorticoid-responsive alveolar epithelial cells A549 [62] were used since endogenous FOXO3 mRNA was also inducible after Dex treatment. Under these conditions, the full-length FOXO3 promoter construct showed the highest induction after Dex treatment. When GRE1 was deleted, induction reproducibly dropped, indicating that this element contributes to glucocorticoid induction of the promoter. In contrast, further truncation of the FOXO3 promoter from approximately −4 kb to −11 bp did not drastically affect the level of glucocorticoid induction, although the potential GRE2 was removed. This indicates that this second potential GRE apparently has no major function in glucocorticoid induction of the FOXO3 promoter driven luciferase reporter. Only when the residual 100 bp of the smallest promoter construct (referred as Not I, Figure 3C) was removed, luciferase activity was no longer increased by Dex, indicating that GRE3 is also involved in glucocorticoid induction of FOXO3 transcription.

To confirm that GR binds directly to these potential GREs within the endogenous FOXO3 promoter upon ligand addition, chromatin immunoprecipitation assays (ChIP) were performed using either untreated or Dex treated MCF-10A cells (Figure 3D). Primers were designed in order to generate PCR products which distinguish between the different GR binding sites within the FOXO3 promoter (Figure 3A; referred as ChIP A, ChIP B and ChIP C). Furthermore, chromatin was sonicated to shear DNA to an average size of <500 bp to exclude cross-detection of GR binding to GRE1 by primers specific for GRE2 and *vice versa* (Figure 3D, upper panel). As depicted in figure 3D, a GR-specific antibody only enriched GRE1 and GRE3 when cells were pre-treated with Dex. In contrast, only a marginal enrichment could be observed when GRE2 was examined.

Taken together, the ChIP analysis confirmed the interaction between GR and GRE1 and 3 of the endogenous FOXO3 promoter upon glucocorticoid treatment. Consistent with the reporter assay, this suggests that enhancement through these elements accounts for the endogenous transcriptional induction of FOXO3 by glucocorticoids.

### *In vivo* Activation of FOXO3 Transcription by Glucocorticoid Treatment

**Figure 4:**
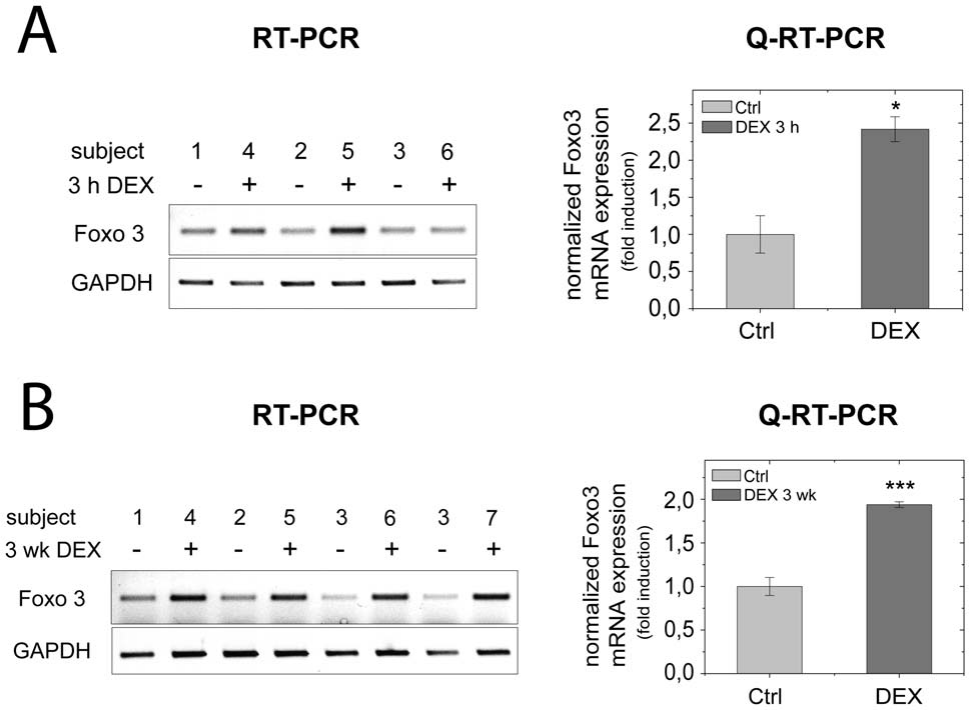
Transcriptional activation of Foxo3 by glucocorticoids in mice. Semi-quantitative (left panel) and quantitative real-time PCR analysis (right panel) of Foxo3 mRNA levels in livers of C57Bl6 mice treated with dexamethasone (DEX) (1 mg/kg/day) or saline (Ctrl) for a period of (**A**) 3 h or (**B**) 3 weeks. Bars represents the means ± standard deviation (n: number of animals; panel A, n = 6; panel B, n = 7).

To exclude that FOXO3 induction by glucocorticoids was only the result of an *in vitro* adaptation effect after long-term cell cultivation, we investigated Foxo3 mRNA expression in the liver of C57/BL6 mice. Semi-quantitative RT-PCR and quantitative real-time PCR revealed the immediate early induction of Foxo3 mRNA expression by 2.5-fold in the liver of animals that were treated with Dex 3 hours before sacrifice (Figure 4A). Foxo3 even remained inducible in chronically treated mice, receiving repeated Dex injections every 24 hours for 3 weeks (Figure 4B). These results indicate that also under *in vivo* conditions Foxo3 mRNA induction can be considered as an immediate early glucocorticoid response that is highly conserved between mouse and human.

### FOXO3 Coordinates LKB1 Gene Expression Depending on the Metabolic Status of the Cell

As shown above, both SGK-1 and FOXO3 are immediate early GR target genes. Conversely, induction of SGK-1 also resulted in a rapid inhibitory post-translational modification (T32 phosphorylation) while concomitantly transcription of FOXO3 was induced (Figure 2B). Consequently, repression of FOXO3 target genes, as shown for LKB1 and others, could be noticed (Figure 2). Since this seemed *prima vista* contradictious, we wondered why glucocorticoids activate FOXO3 transcription but simultaneously impair its activity through SGK-1 induction and succeeding inhibitory phosphorylation.

To get insight in this question, we took into account that glucocorticoids are increasingly produced and released from the adrenal cortex especially under conditions of biological stress [63] and prolonged exercise [64]–[66]. Under these settings, which are normally accompanied by low cellular energy levels, glucocorticoids function as stimulators of gluconeogenesis in the liver [48], [49] in order to restore cellular energy levels by providing glucose to the periphery [50]. Low cellular energy levels are characterized by high AMP/ATP ratios which in turn activate the AMP-activated protein kinase (AMPK) [43]. Since AMPK phosphorylates FOXO3 and thereby activates its transcriptional activity [44], we hypothesized that AMPK activating stimuli can also alter the above described glucocorticoid-mediated SGK-1/FOXO3/LKB1 response circuit.

**Figure 5:**
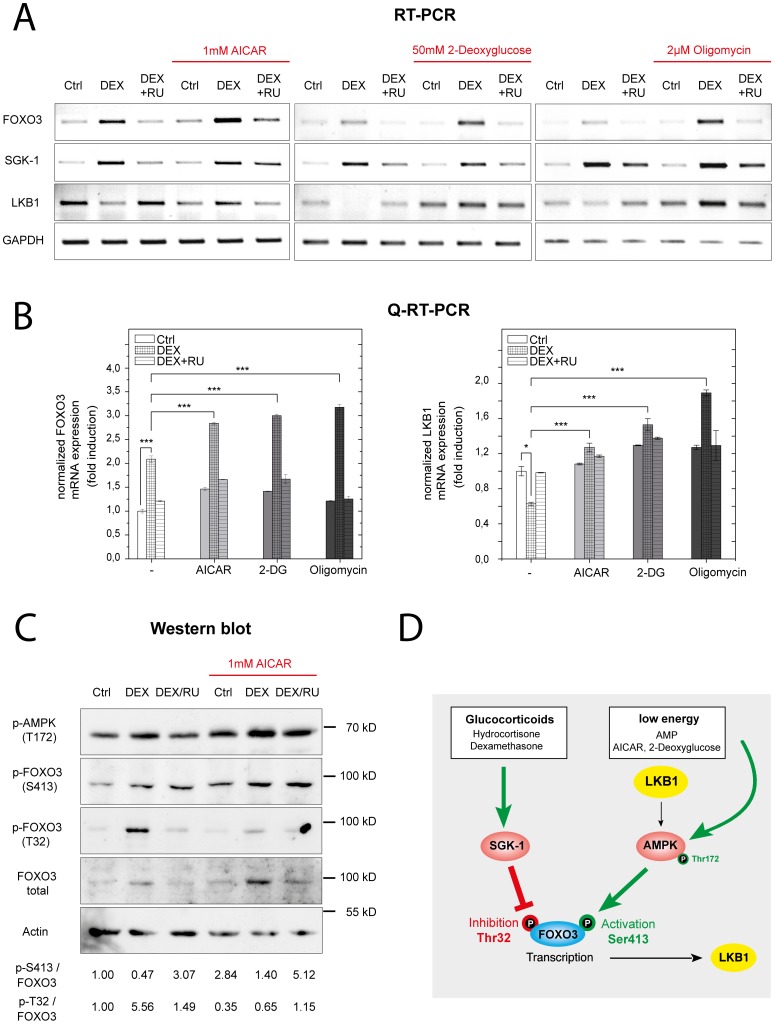
FOXO3 coordinates LKB1 gene expression depending on the metabolic status of the cell. MCF-10A cells were treated with AMPK activating stimuli (1 mM AICAR, 50 mM 2- Deoxyglucose or 2 µM Oligomycin) in combination either with vehicle (Ctrl), 1 µM dexamethasone (DEX) or a mixture of 1 µM dexamethasone and 1 µM RU-486 (DEX/RU) for 18 hours. (**A**) Relative mRNA levels of SGK-1, LKB1, FOXO3 and GAPDH as internal control were analysed by semi-quantitative RT-PCR and separated in a 1% agarose gel after ethidium bromide staining. (**B**) Relative mRNA levels of LKB1 and FOXO3 were analysed by quantitative real time PCR and normalized to GAPDH (mean ±SD, n = 3). (**C**) Western blot analyses of the relative protein levels of phospho-AMPK (T172), phospho-FOXO3 (S413), phospho-FOXO3 (T32), total FOXO3 in comparison to actin as internal control. The ratios of p-FOXO3/FOXO3 for each experimental condition are indicated. The data shown is representative of three independent experiments. (**D**) Schematic illustration underlying the glucocorticoid-dependent modulation of FOXO3 transcriptional activity.

To prove this hypothesis, MCF-10A cells were either treated with Dex alone or in combination with drugs that activate AMPK (Figure 5). Beside AICAR, an adenosine analog that mimics intracellular AMP accumulation and thereby directly activates AMPK [67], we also used drugs that indirectly stimulate AMPK by in fact increasing intracellular AMP levels. While 2-deoxyglucose inhibits glycolysis [68], oligomycin acts as ATP-synthase inhibitor that blocks oxidative phosphorylation [69]. After 18 hours of incubation with Dex in conjunction with these drugs, RNA was isolated and analysed by semi-quantitative RT-PCR (Figure 5A). Notably, although SGK-1 was equally induced - either by Dex alone or in combination with AMPK activating drugs - the impact on LKB1 expression was completely opposed. Although Dex alone repressed LKB1 mRNA, a combination either with AICAR, 2-deoxyglucose or oligomycin induced gene expression. Conversely, concomitant treatment with the GR-antagonist RU-486 mainly prevented both effects. Analysis of LKB1 mRNA levels by quantitative real-time PCR (Figure 5B, right panel) revealed an approximate reduction of 50% after Dex treatment. This was reverted by simultaneous treatment with AICAR, 2-deoxyglucose or oligomycin, leading to induction rates ranging from 20 to 100%, indicating that AMPK activating stimuli can overcome glucocorticoid-induced LKB1 repression.

Since AMPK phosphorylation of FOXO3 on S413 activates its transcriptional activity [44], we hypothesized that the change of the glucocorticoid-induced LKB1 repression into an induced state is caused by a modulation of the FOXO3 phosphorylation status. To examine AMPK-mediated FOXO3 S413 phosphorylation, a phospho-specific antibody was generated and monitored for its specificity by western blot analysis. As shown in figure S1, a strong phosphorylation of FOXO3 upon 2-deoxyglucose addition could be discerned, which was absent when the serine at position 413 was mutated. When Dex/AICAR treated MCF-10A cells were examined, the following picture emerged (Figure 5C): While Dex alone lead to an increased FOXO3 phosphorylation at T32 that resulted in LKB1 repression (Figure 5A and B), a combination with an AMPK activator abrogated inhibitory FOXO3 phosphorylation at T32 despite SGK-1 expression still remained induced (Figure 5A). Instead, an increasing phosphorylation of FOXO3 at the AMPK phosphorylation site S413 could be discerned. This corresponds with enhanced AMPK activity as monitored by T172 phosphorylation [38] and an induced LKB1 transcription (Figure 5A and B). Note that SGK-1 mediated FOXO3 T32 phosphorylation was not only abrogated by AICAR, but also by concomitant treatment with 2-deoxyglucose (Figure S2). A schematic overview of the processes, underlying the glucocorticoid-dependent modulation of FOXO3 transcriptional activity, is shown in figure 5D and can be summarized as follows:

When cells reacted to metabolic stress, characterized by low intracellular energy and high glucocorticoid levels [50], [64]–[66], the transcription factor FOXO3 was immediately activated on the transcriptional level and phosphorylated by AMPK at position S413. This resulted in an increased transcription of the FOXO3 target gene LKB1. Under these conditions, induced SGK-1 cannot efficiently phosphorylate and inhibit FOXO3 activity. In other words, SGK-1 can apparently only inactivate FOXO3 when intracellular energy levels were high, indicating a hierarchical order within the signalling network, in which low energy has the highest priority. Hence, FOXO3 may function as an anabolic-catabolic switch, coordinating target gene expression depending on the metabolic status of the cell.

### Glucocorticoid-induced Transcriptional Activation of FOXO3 is Further Enhanced by AMPK Activating Stimuli – requirement of *“de novo”* Protein Synthesis

**Figure 6:**
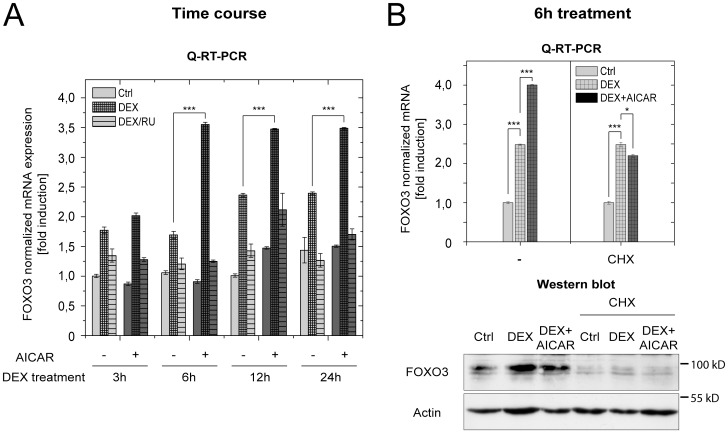
Glucocorticoid-induced transcriptional activation of FOXO3 is further enhanced by AMPK activation. (A) MCF-10A cells were treated with 1 mM of specific AMPK activator AICAR in combination with either vehicle (Ctrl), 1 µM dexamethasone (DEX) or a mixture of 1 µM dexamethasone and 1 µM RU-486 (DEX/RU) for 3, 6, 12 and 24 h. (B) MCF-10A cells were treated with vehicle (Ctrl), 1 µM dexamethasone (DEX) or a combination of 1 µM dexamethasone and 1 mM AICAR (DEX+AICAR) in the presence or absence of 10 µg/ml cycloheximide (CHX) for 6 h. Relative mRNA levels of FOXO3 were analysed by quantitative real-time PCR (mean ±SD, n = 3). Relative protein levels of FOXO3 in comparison to actin as internal control were analysed by western blotting in the presence or absence of cycloheximide.

FOXO3 expression was not only immediately induced by glucocorticoids, but even further enhanced when low energy status was induced (Figure 5). To further investigate this effect, we performed a time course analysis of FOXO3 mRNA expression after GR treatment and AMPK activation using quantitative real-time PCR (Figure 6A). After 3 hours, both, Dex alone or in combination with AICAR, induced FOXO3 mRNA expression to the same extent (app. 1.75 to 2-fold). However, after 6 hours, induction of FOXO3 expression by the combination of Dex and AICAR was already higher (3.5 fold) than the induction by Dex alone (1.75 fold) and remained elevated over time. Under both conditions, FOXO3 induction was abrogated by simultaneous treatment with the GR antagonist RU-486. Consistent with the observation that AICAR treatment alone was not sufficient to effectively induce FOXO3 mRNA, these results suggest that GR and AMPK cooperate to activate FOXO3 transcription. Since enhanced FOXO3 mRNA induction after Dex/AICAR treatment was temporally delayed, we assumed that *de novo* protein synthesis is required to mediate this effect.

To prove this assumption, induction of FOXO3 transcripts was compared in the presence or absence of ongoing protein synthesis. MCF-10A cells grown in medium containing 2% charcoal stripped FBS were pre-treated for 30 min with or without 10 µg/ml cycloheximide and then incubated with the indicated combinations of 1 µM Dex and 1 mM AICAR for 6 h. FOXO3 mRNA levels were than analysed by quantitative real-time PCR. As depicted in figure 6B, the presence of cycloheximide abrogates the enhancement of FOXO3 induction, corroborating the notion that *de novo* protein synthesis is indispensable to enhance FOXO3 transcription upon combined Dex/AICAR treatment.

Interestingly, the enhanced induction of FOXO3 transcripts that appeared after combined Dex/AICAR treatment strongly correlates with the post-translational activity of FOXO3 itself (Figure 5 and 6). Either when FOXO3 proteins were post-translationally inhibited by SGK-1 after exclusive Dex treatment, or when cycloheximide inhibited *de novo* synthesis of FOXO3 proteins (Figure 6B), transcriptional induction of the FOXO3 gene was only half as pronounced as under the combined Dex/AICAR conditions, were FOXO3 was entirely post-translationally active (Figure 6; approx. only 2-fold instead of 4-fold). This suggests that *de novo* synthesized FOXO3 molecules themselves could be responsible for the temporally delayed enhanced transcriptional induction of their own gene under low energy conditions.

### FOXO3 Binds to its Own Promoter and Stimulates its Expression via a Positive Autoregulatory Feedback Loop in Combination with the Glucocorticoid Receptor

**Figure 7:**
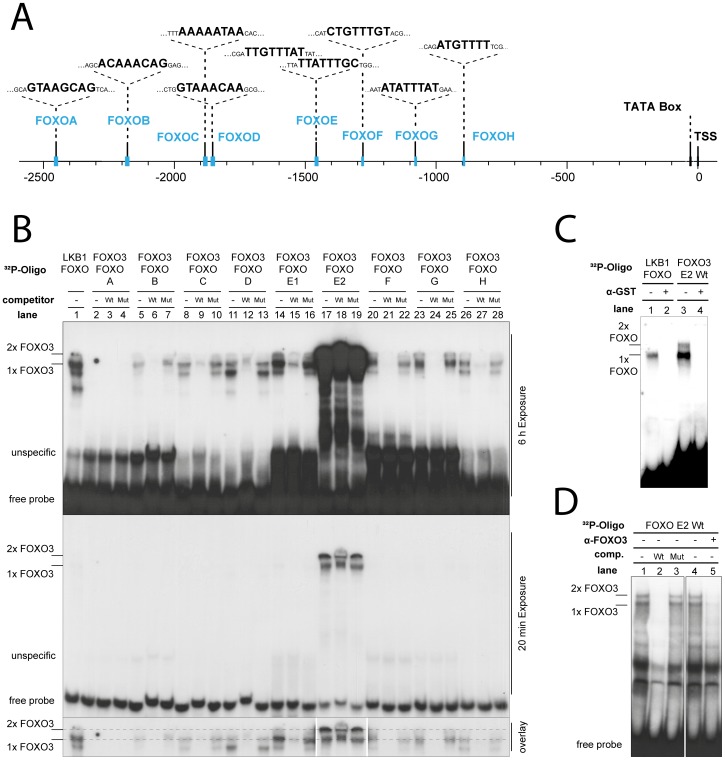
FOXO3 binds to its own promoter. (**A**) Illustration of the FOXO3 promoter region extending from nucleotide position -2500 to +91 relative to the transcription start site. Positions of the TATA box, the transcription start site (TSS) and potential FOXO3 binding sites (FOXO A-H) as well as their sequences are indicated. (**B**) ^32^P-labeled double-stranded oligonucleotides containing the FOXO3 binding sites of the LKB1 promoter (LKB1 FOXO) or the different potential FOXO3 binding sites of the FOXO3 promoter (FOXO3 FOXO A-H) were incubated with 25 ng of recombinant GST-tagged FOXO3 protein, separated in a 4% polyacrylamide gel and visualized by autoradiography after 6 h (upper panel) or 20 min (lower panel) exposure. Formation of sequence specific protein complexes was confirmed by competition with a 500-fold molar excess of unlabeled oligos containing either the wild-type (Wt) or the mutant (Mut) FOXO3 site of the LKB1 promoter. Complexes containing either one (1x FOXO3) or two FOXO3 complexes (2x FOXO3) are marked. (**C**) Complex formation between GST-FOXO3 and ^32^P-labeled double-stranded oligonucleotides containing the FOXO3 binding sites of the LKB1 promoter (LKB1 FOXO), the wild-type E2-binding site of the FOXO3 promoter (FOXO3 E2 Wt). Addition of a GST specific antibody (α-GST, lanes 2 and 4) confirms that the composition of protein-DNA complexes consists of FOXO3 molecules. (**D**) Endogenous complex formation between 4 µg of nuclear extracts from MCF-10A cells and the FOXO3 E2 wild-type site in the presence of unlabeled wild-type E2 (Wt, lane 2) or mutant E2 (Mut, lane 3) competitor. Addition of a FOXO3 specific antibody (α-FOXO3, lane 5) confirms that the composition of protein-DNA complexes consists of endogenous FOXO3 molecules.

Since the expression of the closely related FOXO1 and FOXO4 genes is stimulated by FOXO3 [70], we reasoned that under low energy conditions, FOXO3 may also stimulate its own promoter in an autoregulatory manner, an effect that is apparently impeded by cycloheximide treatment (Figure 6B). Indeed, monitoring the genomic sequence upstream of the human FOXO3 gene (Figure 7A), eight potential FOXO3 binding sites (FOXO A–H) with close homology to the consensus core binding motive (5′-[A/G]TAAA[C/T]AA-3′) [71] were detected (Table 1). To test whether FOXO3 binds to these predicted sequences, electrophoretic mobility shift assays (EMSAs) were performed (Figure 7B). Purified GST-tagged FOXO3 protein interacted specifically with an oligonucleotide, containing the FOXO3 consensus site from the LKB1 promoter [33] (lane 1) as well as with the oligonucleotides referred as B, C, D, E1, E2, F, G and H of the FOXO3 promoter (lanes 5–28). In contrast, no interaction was found between FOXO3 and site A of the FOXO3 promoter (lanes 2–4). Sequence specificity of the binding was further confirmed by competition with an unlabeled oligonucleotide, containing either the wild-type FOXO3 consensus site from the LKB1 promoter (WT, lanes 3, 6, 9, 12, 15, 18, 21, 24, 27) or the corresponding mutant (Mut, lanes 4, 7, 10, 13, 16, 19, 22, 25, 28).

**Table 1:** Oligonucleotides used in EMSA.

Oligo	Sequence	Position relative to TSS
LKB1 FOXO Wt	5′-GGGGAGGGAG**GTAAACAA**GATGGCGGC-3′	−27 to −1
LKB1 FOXO Mut	5′-GGGGAGGGAG**GTAGCCAA**GATGGCGGC-3′	−27 to −1
FOXO3 FOXO A	5′-AGCCACCGCA**GTAAGCAGT**CAGAGCCC-3′	−2439 to −2413
FOXO3 FOXO B	5′-TGGGCGAAGC**ACAAACAG**GAGGCTTTG-3′	−2168 to −2142
FOXO3 FOXO C	5′-CATTCCATTT**AAAAATAA**CACCCACGT-3′	−1865 to −1839
FOXO3 FOXO D	5′-ACGTGCGCTG**GTAAACAA**GCGCGCGCG-3′	−1842 to −1816
FOXO E1	5′-TTTACTCGA**TTGTTTATTATTTGC**TGG-3′	−1446 to −1420
FOXO E2	5′-GA**TTGTTTATTATTTGC**TGGGGGGCGG-3′	−1439 to −1413
FOXO F	5′-TGACATCAT**CTGTTTGT**ACGCGTCGAA-3′	−1270 to −1244
FOXO G	5′-TTAAAAAAT**ATATTTAT**GAACTAGTGT-3′	−1059 to −1033
FOXO H	5′-CTTGTGCAG**ATGTTTTT**CGTTTAACCT-3′	−949 to −923
FOXO3 FOXO E2 WT	5′-TAGAATTTTACTCGA**TTGTTTATTATTTGC**TGGGGGGCGG-3′	−1452 to −1413
FOXO3 E2 Linker	5′-GA**TTGTTTAT** TAGCGCGGCGCG**TTATTTGC**TGGGGGGCGG-3′	−1452 to −1413
FOXO3 E2 MUT	5′-TAGAATTTTACTCGA**TTGCGTATTACGTGC**TGGGGGGCGG-3′	−1452 to −1413

FOXO3 core binding motive is indicated in bold. Parts of adjacent FOXO3 binding sites within the same oligonucleotide are underlined.

Of note was the finding that complex formation between FOXO3 and the E2 site was tremendously stronger than complex formation with the corresponding site of the LKB1 promoter (compare lane 1 and 17). Furthermore, binding competition to the E2 site was rather weak, indicating that FOXO3 has a higher affinity to the E2 site within the FOXO3 promoter than to the FOXO3 site within the LKB1 promoter. This was further confirmed by other competition experiments (Figure S3). While binding of FOXO3 to the E2 site was completely inhibited using 50 nM of unlabeled E2 wild type oligo, 200 nM of the FOXO site derived from the LKB1 promoter was necessary to achieve the same effect.

A unique feature of the E2 site is that two FOXO3 binding sites are located in close proximity to each other (Figure 7A). In contrast to the FOXO3 sequence within the LKB1 promoter, where only a single complex is formed (referred as “1x FOXO”, Figure 7), the E2 site of the FOXO3 promoter can also produce a slower migrating FOXO3 complex (referred as “2x FOXO”, Figure 7). Both of them can be disrupted by the addition of a GST specific antibody confirming specific binding of GST-FOXO3 molecules (lanes 2 and 4, Figure 7C).

To analyse whether endogenous FOXO3 can also bind to the FOXO3 promoter, nuclear extracts from MCF-10A cells were incubated with the E2 probe (Figure 7D). Here, the two uppermost complexes were disrupted by a wild-type, but not by a mutated E2, indicating that the interaction is sequence specific. These two complexes were also disrupted by the addition of a FOXO3 specific antibody but not by an unspecific IgG control (compare lane 4 and 5, Figure 7D), confirming the authenticity of FOXO3 binding.

**Figure 8:**
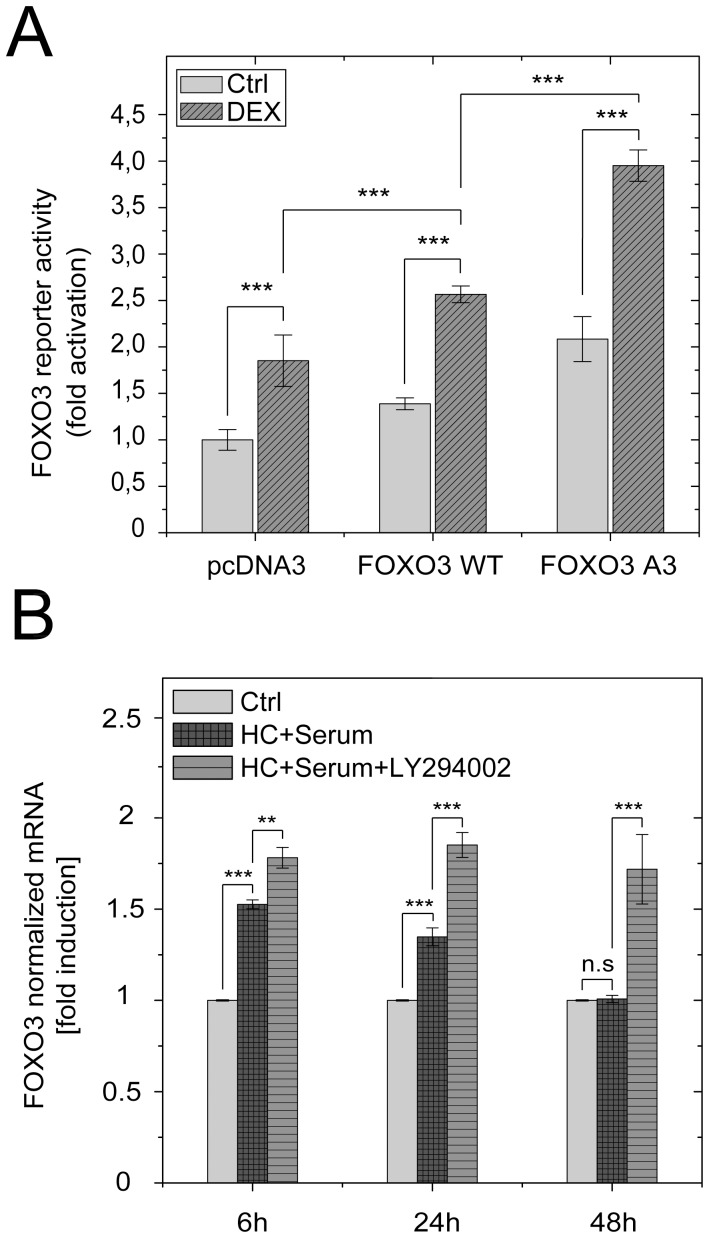
FOXO3 activates its own promoter via a positive autoregulatory feedback loop in combination with GR. (**A**) FOXO3 reporter activity in A549 cells after treatment with either vehicle (Ctrl, light grey) or 1 µM dexamethasone (DEX, dark grey) for 12 h. Reporter activity is expressed as fold of luciferase activity (relative light units normalized to renilla luciferase activity) obtained from co-transfection of the plasmid containing the full length FOXO3 promoter (nucleotides −4255 to +91) together with the empty expression vector (pcDNA3) without dexamethasone treatment. Instead of the empty vector the same amount of either a FOXO3 wild-type expression plasmid (FOXO3 WT) or a SGK-1 phosphorylation site deficient triple mutant (T32A/S253A/S315A) of FOXO3 (FOXO3 A3) was co-transfected. Each bar represents the means ± standard deviation of a triplicate experiment. (B) Quantitative real-time PCR analysis of FOXO3 mRNA levels in MCF-10A cells which were starved of growth factors in medium containing 2% of charcoal stripped FBS for 72 h (Ctrl), transferred into normal growth medium containing 10 µg/ml human insulin and 5% horse serum and stimulated with 0.5 µg/ml hydrocortisone (HC+Serum) for 6, 24 and 48 h, in the presence or absence of 50 µM of the specific PI3-kinase inhibitor LY294002.

In order to test whether FOXO3 could activate the expression of its own gene in the presence of glucocorticoids, we co-transfected the FOXO3 promoter driven luciferase reporter together with a plasmid encoding either wild-type FOXO3, or a SGK-1 phosphorylation site deficient mutant FOXO3 A3 into A549 cells. After 12 hours of incubation with or without Dex, luciferase activity was measured (Figure 8A). Consistent with our previous experiments (also see Figure 3C), Dex treatment induced FOXO3 promoter activity. This effect was further pronounced when both FOXO3 expression plasmids were delivered suggesting that GR and FOXO3 cooperate in activating the FOXO3 promoter. In particular the combination of Dex and the ectopic delivery of the PKB/SGK-1 phosphorylation site deficient mutant FOXO3 A3, which is resistant to growth factor inactivation, induced the FOXO3 promoter to a greater extent than Dex alone.

Since both negative regulators of FOXO3, PKB and SGK-1 are downstream effectors of the PI3-kinase [26]–[28], we investigated whether PI3-kinase activation upon growth factor treatment decreases the glucocorticoid-driven induction of FOXO3 gene expression or whether inhibition of the PI3-kinase pathway further enhances transcription. This would further support the notion of a positive auto-regulatory feedback loop in which FOXO3 activates the expression of its own gene, a process that would be negatively regulated by PI3-kinase activation.

To analyse the consequence of PI3-kinase activation on glucocorticoid induced FOXO3 transcription, we performed a time course experiment with the natural glucocorticoid hydrocortisone in growth medium containing the PI3-kinase activating stimuli insulin and serum. In detail, MCF-10A cells were first starved from growth factors in medium containing 2% of charcoal stripped FBS for 72 hours, transferred into normal growth medium containing 10 µg/ml human insulin and 5% horse serum, and stimulated with 0.5 µg/ml hydrocortisone for 6, 24 and 48 hours in the presence or absence of 50 µM of the specific PI3-kinase inhibitor LY294002. Then, FOXO3 mRNA levels were analysed by quantitative real-time PCR (Figure 8B).

While the hydrocortisone mediated induction of FOXO3 transcripts is diminished in the presence of serum in a time-dependant manner, returning to basal levels after 48 h, decrease was prevented by inhibition of the PI3- kinase pathway (Figure 8B). This supports the notion that PI3-kinase activation reduces glucocorticoid-driven induction of FOXO3 gene expression, supporting a mechanism by which FOXO3 activates the expression of its own gene. Such a process, which is negatively influenced by PI3-kinase activation, further increases glucocorticoid-induced FOXO3 expression and suggests that this kind of enhancement also accounts for the endogenous transcriptional induction by the combination of glucocorticoids and low energy under natural physiological conditions.

### AMPK is Essential for FOXO3 Induced Activation of LKB1 and FOXO3 Transcription

**Figure 9:**
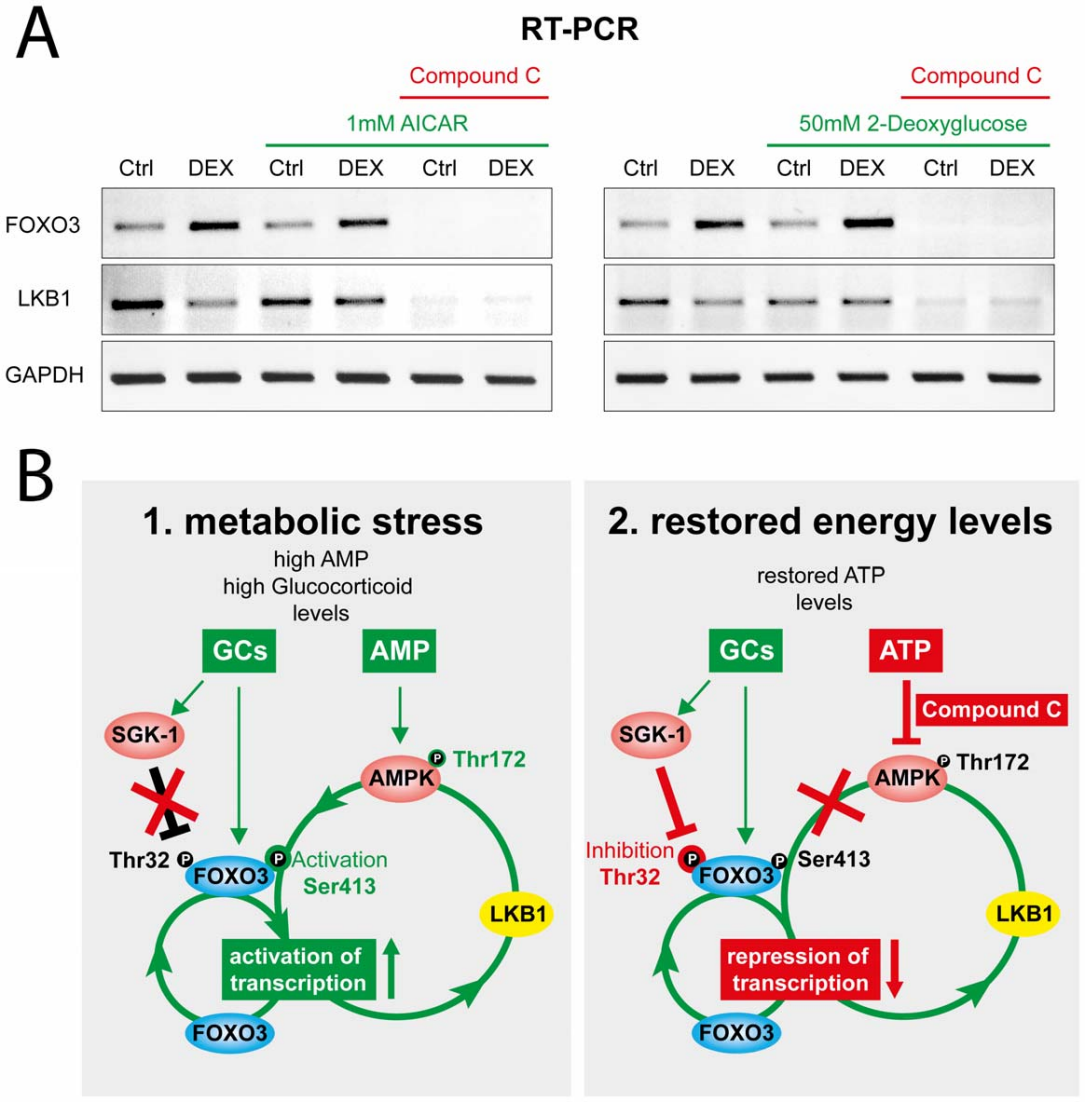
AMPK is essential for FOXO3 induced activation of LKB1 and FOXO3 transcription. (**A**) MCF-10A cells were either treated with vehicle (Ctrl) and 1 µM dexamethasone (DEX) or in combination with either 1 mM AICAR (left panel) or 50 mM 2-deoxyglucose (right panel) in the presence or absence of 20 µM of the specific AMPK inhibitor “Compound C” for 18 h. Relative mRNA levels of LKB1, FOXO3 and GAPDH as internal control were analysed by semi-quantitative RT-PCR in a 1% agarose gel after ethidium bromide staining. The data shown is representative of three independent experiments. (**B**) Illustration of metabolic regulation of FOXO3’s transcriptional activity and subsequent effects on FOXO3 and LKB1 expression. Activating events are indicated in green colour, inhibiting events in red. High glucocorticoid levels immediately activate FOXO3 and SGK-1. Following stimulation of FOXO3 transcription, the protein is phosphorylated at threonine 32 (Thr 32), leading to its inactivation. Treatment with AMPK activating stimuli (metabolic stress) triggers phosphorylation of FOXO3 by AMPK at position 413 of the serine residue (Ser 413), thereby counteracting inactivation of FOXO3 by SGK-1. This results in a temporally delayed further enhancement of FOXO3 transcription since the FOXO3 protein can bind and activate its own gene promoter via a positive autoregulatory feedback loop in the presence of glucocorticoids. Functional FOXO3 in turn induces the master upstream kinase that further actives AMPK by threonine phosphorylation at position 172 (Thr 172). Restored ATP levels or inhibition of AMPK by compound C interrupts this circuit.

In the experiments described above, we have shown that both FOXO3 and LKB1 are FOXO3 target genes that are activated by a combined action of glucocorticoids and low energy. To prove whether AMPK-mediated activation of FOXO3 was responsible for the induced target gene expression, we analysed the effect of AMPK inhibition on the induction of both genes. For this purpose, the relative effects of Dex alone and under low energy conditions were compared in the presence or absence of the AMPK specific inhibitor “compound C” [72]. MCF-10A cells grown in medium containing 2% charcoal stripped FBS were pre-treated for 1 hour with or without “compound C” and then incubated with the indicated combinations of 1 µM Dex and 1 mM AICAR or 50 mM 2-deoxyglucose for 18 hours. FOXO3, LKB1 and GAPDH mRNA levels were than analysed by semi-quantitative RT-PCR (Figure 9A). Consistent with the previous experiments, Dex-mediated repression of LKB1 expression was abrogated, and FOXO3 transcription was further induced via concomitant AMPK activation by AICAR or 2-deoxyglucose. In contrast, when AMPK was inhibited, FOXO3 as well as LKB1 transcription was strongly reduced. These results confirm an essential role of AMPK in FOXO3 target gene activation and further support a central function of FOXO3 in coordinating target gene expression depending on the metabolic status of the cell (Figure 9B).

## Discussion

FOXO3 transcription factor regulates a diverse array of physiological processes and has been functionally linked to cell cycle arrest [5], [6], [32], DNA damage repair [7], [8], apoptosis [9], [10], [25], oxidative stress-response [20]–[22] and energy metabolism [17]–[19], [73]. While the majority of FOXO3 studies relates to its post-translational control by acetylation, ubiquitination and phosphorylation [74], transcriptional regulation of FOXO3 has been less well studied. To address this gap in understanding, the aim of the present report was to determine how metabolic hormones and low cellular energy influence FOXO3 gene expression and subsequently FOXO3 target gene transcription.

The data obtained in this study and their potential relevance in coordinating the energy balance is schematically overviewed in figure 9B: when cells reacted to high glucocorticoid levels, FOXO3 as well as SGK-1 were immediately activated on the transcriptional level (Figure 2, 4 and 5, see panel B, left). Induction of FOXO3 transcripts required GR-binding steroids and was reversed by concomitant treatment with the GR antagonist RU-486 (Figure 2). Consistent with the stimulation of FOXO3 being a primary response to glucocorticoids, analysis of genomic DNA as well as ChIP assays revealed three apparent GREs in the FOXO3 promoter. Indeed, the expression of a luciferase reporter gene was stimulated by Dex when fused to the FOXO3 promoter. Additionally, when metabolic stress was simulated by concomitant treatment with AMPK activating stimuli, rapid phosphorylation of FOXO3 by AMPK prevented the inactivation of FOXO3 by SGK-1 (Figure 5). This again resulted in a temporally delayed enhanced induction of FOXO3 transcription (Figure 6A), which was depending on *de novo* protein synthesis (Figure 6B). In line with the observations that glucocorticoid-mediated induction of FOXO3 transcripts was PI3-kinase sensitive (Figure 8), and with the finding that FOXO3 could actively bind and activate its own gene promoter (Figure 7), these results indicate that FOXO3 activates its own expression via a positive autoregulatory feedback loop in the presence of glucocorticoids.

In conclusion, these findings unravelled a mechanism by which glucocorticoids and low energy can acutely stimulate FOXO3 activity, providing a molecular basis for how FOXO3 mediates effects of both, catabolic hormones, and insulin on energy metabolism. Under these conditions FOXO3 acts as an anabolic-catabolic switch, coordinating target gene expression depending on the metabolic status of the cell (Figure 9B). Consistent with this idea is that FOXO transcription factors are activated in response to the fasting hormone glucagon [19], which has partially similar effects on glucose metabolism than glucocorticoids [45], [47]–[49]. Although both hormones activate gluconeogenesis in the liver [47]–[49], glucagon differs from glucocorticoids in activating FOXO protein post-translationally through deacetylation [19]. In this context, both hormones, which are released under similar metabolic conditions [50], [64]–[66], might cooperate in activating FOXO3 target gene expression by combining transcriptional and post-translational levels of activation.

In addition to hormone activation of FOXO3 and in agreement with our study, recent evidence indicates that FOXO3 is also activated by energy deprivation through AMPK phosphorylation [44]. In addition to this well characterized post-translational activation of FOXO3, which seems to be evolutionary conserved [75], [76], we find that FOXO3 is also activated on the transcriptional level after AMPK activation. Especially the combination of glucocorticoids and AMPK activating stimuli (e.g. AICAR or 2-deoxyglucose) activated FOXO3 transcription most efficiently (Figure 5 and 6). Indeed, recent findings showed that AMPK activation upon sepsis or AICAR injection increased FOXO3, FOXO1 but not FOXO4 mRNA levels in skeletal muscle of mice [77]. In contrast, the same study reports a decrease of all FOXO mRNA levels in C2C12 myotubes after AMPK activation using AICAR or metformin [77].

Therefore, analysis of the physiological function of low energy mediated FOXO3 induction in cells that senses changes in energy levels like, for instance, hepatocytes, adipocytes or pancreatic β-cells, will be one of the future goals. Under conditions of metabolic stress, the enhanced transcriptional activity of FOXO3 also increased the expression of the FOXO3 target gene LKB1 [33] (Figure 5), which in turn can phosphorylate and thereby activate AMPK [37], [38]. Induction of LKB1 gene expression by FOXO3 might ensure a sufficient activation of AMPK under conditions of metabolic stress. Once activated, AMPK switches off anabolic pathways while simultaneously activating catabolic pathways, which result in the production of ATP [43]. When cellular energy levels are restored, AMPK is allosterically inactivated by ATP [41], [42]. Under such conditions, in which energy levels were restored, the induced SGK-1 could phosphorylate and thereby inactivate FOXO3s transcriptional activity, resulting in a subsequent repression of LKB1 gene expression (Figure 9B, see schematic overview). Although AMPK is mainly allosterically regulated by AMP/ATP ratios, the repression of the FOXO3 target LKB1 might contribute to a sustained inactivation of AMPK when energy levels are restored.

The fact that the glucocorticoid-mediated induction of FOXO3 transcripts was enhanced by AMPK activation (Figure 5 and 6), but inhibited by the PI3-kinase (Figure 8), illustrates a novel way in which the PI3-kinase-PKB and the LKB1-AMPK pathways have opposite biological functions. While the PI3-Kinase-PKB pathway stimulates cell proliferation and survival and thereby promotes tumour growth [78], the LKB1-AMPK pathway promotes cell cycle arrest and tumour suppression [79]. FOXO3 is one of the intersections between the two pathways, being post-translationally inhibited by downstream effectors of the PI3-kinase [25], [29] and post- translationally activated by AMPK [44]. In addition to this well characterized post-translational regulation, our data show that similar regulations also occur on the transcriptional level of FOXO3. Thus, the PI3-kinase and the LKB1-AMPK signalling pathways may coordinate a series of transcriptional and post-transcriptional changes that allow cells to adapt to changes in the energy status.

A cross-talk between the PI3-kinase and the LKB1-AMPK might also play a critical role in the pathogenesis of cancer. LKB1 is a classical tumour suppressor that is mutated in the inherited Peutz-Jeghers cancer syndrome [80], [81] and that is also frequently inactivated in a large percentage of sporadic lung and cervical carcinomas [35], [36]. Similarities between the Peutz-Jegers and the Cowden disease [82], a cancer syndrome caused by mutations in the PI3-kinase inhibitor PTEN, further support a link between the two pathways. Also, deletion of all FOXO1, FOXO3, and FOXO4 alleles in adult mice induced a cancer prone phenotype, supporting a tumour suppressing function of these proteins. One way by which LKB1 and FOXO proteins could execute tumour suppression is by activating each other. For this reasons, a cross-talk between the PI3-kinase and the LKB1-AMPK pathway at the level of FOXO3 regulation might play a critical role in carcinogenesis.

## Materials and Methods

### Antibodies

Mouse monoclonal anti-LKB1 (ab15095) was obtained from Abcam, rabbit monoclonal anti-phospho-AMPKα Thr172 (2535/40H9) and rabbit polyclonal phospho-FOXO3 Thr32 (9464) were purchased from Cell Signaling Technology. Rabbit polyclonal anti-FOXO3 (sc-11351 X), rabbit polyclonal anti GR (sc-1004), rabbit polyclonal anti glutathione-S-transferase (GST) (sc-459) and normal rabbit IgG (sc-2027) were from Santa Cruz Biotechnology. Horseradish peroxidase-conjugated secondary antibodies, polyclonal goat anti-rabbit IgG (W4011) and anti-mouse IgG (W4012) were obtained from Promega. For the generation of the polyclonal rabbit anti-human phospho-FOXO3 Ser413 antibody, the phospho-peptide PPSQPSPTGGLMQRSS(pS)FPYT was synthesized by solid-phase technique using the fluoren-9-ylmethoxycabonyl (Fmoc) strategy on a Syro II synthesizer (MultiSynTech, Witten, Germany). The peptide was purified by preparative reversed phase HPLC, analysed by mass spectrometry (using a MALDI time-of-flight system; Reflex IV, Bruker Daltonics, Bremen, Germany), coupled to KLH and injected into a rabbit. A second and a third injection was performed 4 and 6 weeks after the first injection. Two months after the first injection a serum sample was taken and affinity purified against the phosphopeptide. Specificity of the antibody in western blot application was confirmed using lysates from HEK 293 cells over-expressing either wild-type FOXO3 or mutant FOXO3 S413A, which were treated with or without lambda-phosphatase (New England Biolabs) (Figure S1). Either lambda phosphatase treatment or mutation of Ser 413 to Ala completely inhibited the detection confirming that the antibody recognizes FOXO3 only when phosphorylated at S413.

### Plasmids

The plasmids pcDNA3.1 (Invitrogen), pCR-BLUNT (Invitrogen), pGL3-Basic (Promega), pRLSV40 (Promega), the expression plasmid of human full length SGK-1 (Clone ID: 3459056, Thermo Scientific Open Biosystems) and the bacterial artificial chromosome (BAC), containing the human FOXO3 locus on chomosome 6 (Clone ID: RP11-654F13, Source BioScience) have been purchased from commercial suppliers. Expression plasmids of human FOXO3 and FOXO3 A3 were generated as described previously [33]. Briefly, PCR amplified FOXO3 cDNA was cloned into the EcoRV site of the pcDNA3.1 vector. FOXO3 A3 was created by site directed mutagenesis of the three SGK-1 phosphorylation sites T32, S253 and S315 to A. The LKB1 promoter driven luciferase reporter construct (position -2537 to +727 relative to the transcription start site) was constructed as described [33]. The 5′-flanking region of the FOXO3 coding sequence encompassing nucleotides -2313 to +370 relative to the transcription start site was amplified by PCR using 400 nM forward (5′- GGCATTCGGTACAAGGAAGCGAGGACACAGAG -3′) and reverse (5′- CGCTGCTGCCATCTTGACAGTTTCC -3′) primers, 0.2 ng/µl of the pBAC (RP11-654F13) as a template and 1 Unit Phusion High-Fidelity DNA-polymerase (Finnzymes) in a final volume of 25 µl 1x GC-Phusion reaction buffer supplemented with 0.2 mM dNTP’s, 4% of dimethylsulfoxide and 2% of formamide. The PCR-product was purified and cloned into the pCR-BLUNT plasmid using the pCR-BLUNT Cloning Kit (Invitrogen) according to the manufacturer’s instructions. The FOXO3 promoter (position −2313 to +91) was then subcloned into the SmaI site of the pGL3-Basic luciferase reporter vector using AfeI and EcoRV restriction enzymes (New England Biolabs). This construct was termed “FOXO3 ProI”. A second PCR product encompassing nucleotides −4255 to −2235 relative to the FOXO3 transcription start site was amplified as described above using the forward primer (5′-CAGCTCATAGCTGTTTGGTACACGGTGTTCAA-3′) and the reverse primer (5′-CACAAGCTGCACACACTCACTGGCACACATAC-3′), cloned into the pCRBLUNT vector and subcloned into the “FOXO3 ProI” construct using EcoRI and SnaBI restriction enzymes. The resulting FOXO3 promoter driven luciferase construct designated as “FOXO3 Pro full length” contains nucleotides −4255 to +91 relative to the transcription start site. DNA-sequencing confirmed that amplified parts of the FOXO3 locus are identical to the deposited sequence of chromosome 6 on GenBank (accession: NC_000006.11). Deletion constructs of “FOXO3 Pro full length” were generated by restriction digestion, followed by religation using the indicated restriction enzymes (Figure 3A). All plasmids used in transient transfections were purified with the QIAGEN Plasmid Maxi Kit (Qiagen) and verified by sequencing.

### Cell Culture and Drug Treatment

The human embryonic kidney cell line HEK 293, the human alveolar adenocarcinoma cell line A549 as well as the non-tumorigenic mammary epithelial cell line MCF-10A were obtained from American Type Culture Collection. HEK 293 and A549 cells were maintained in Dulbecco’s modified Eagle medium (DMEM) (Sigma) supplemented with 10% (v/v) of foetal bovine serum (FBS) (Invitrogen), penicillin (final concentration: 100 U/ml, Invitrogen) and streptomycin (final concentration: 0.1 mg/ml, Invitrogen), unless otherwise indicated. MCF- 10A cells were cultured in a 1∶1 mixture of DMEM (Invitrogen) and Ham’s F12 (Invitrogen) supplemented with 0.5 µg/ml hydrocortisone (Sigma), 20 ng/ml human epidermal growth factor (Invitrogen, Cat. No.: PHG0311L), 100 ng/ml cholera toxin (Sigma, Cat. No.: C8052- 5 MG), 10 µg/ml human insulin (Sigma, Cat. No.: I2643-50 MG) and 5% horse serum (Invitrogen). For drug treatment, MCF-10A cells were transferred to a 1∶1 mixture of DMEM and Ham’s F12 supplemented with 2% of charcoal stripped FBS (Invitrogen, Cat. No.: 12676011) and subjected to 96 h of steroid hormone withdrawal, followed by treatment with either vehicle (0.1% ethanol), 1 µM dexamethasone (Sigma), 1 µM RU-486 (Cayman Chemicals), 1 mM AICAR (Sigma), 50 mM 2-Deoxyglucose (Sigma), 2 µM Oligomycin (Cell Signalling), 20 µM of AMPK inhibitor Compound C (Merck) or concomitant treatment with the indicated combinations for 18 h, unless otherwise indicated.

### Luciferase Assays

For the LKB1 promoter driven luciferase assays, HEK 293 cells were plated at a density of 1.5×10^6^/10 cm dish in DMEM containing 2% charcoal stripped FBS one day before transfection. Cells were transfected with 500 ng/dish of LKB1-promoter firefly luciferase reporter and 1 ng/dish of pRL-SV40 renilla luciferase plasmid for normalization, 750 ng/dish of the indicated FOXO3 expression plasmid or the corresponding empty vector (pcDNA3.1) and 750 ng/dish of SGK-1 expression plasmid or the corresponding empty vector (pcDNA3.1) using 50 µl/dish of Effectene transfection reagent (Qiagen), 16 µl of Enhancer (Qiagen) and 300 µl of EC buffer (Qiagen) in 10 ml of DMEM containing 2% charcoal stripped FBS. 24 h after transfection cells were lysed in Passive Lysis Buffer (Promega), ectopic expression of FOXO3 was confirmed by western blotting, firefly luciferase activity was analysed and normalized to renilla luciferase activity using the dual-luciferase reporter assay system (Promega) according to the manufacturer’s instructions. For FOXO3 promoter driven luciferase assays, A549 cells were plated at a density of 4×10^4^/well on a white Nunclon F-96-well plate (Nunc, Roskilde, Denmark) in DMEM supplemented with 2% charcoal stripped FBS one day before transfection. Cells were transfected with 200 ng/well of the indicated FOXO3-promoter pGL3 firefly luciferase reporter and 1 ng/well of pRL-SV40 renilla luciferase plasmid for normalization using 1.2 µl/well of Lipofectamine 2000 (Invitrogen) in 150 µl/well of OptiMEM (Invitrogen). 6 h after transfection, medium was changed to DMEM containing 2% of charcoal stripped FBS and cells were treated with dexamethasone or vehicle (0.1% ethanol) for 12 h. 18 h after transfection, cells were lysed and luciferase activity was analysed as described above. For co-expression of FOXO3 and FOXO3 A3 transcription factors and the FOXO3-luciferase reporter, cells were transfected with 100 ng/well of full length FOXO3 promoter driven pGL3 firefly luciferase reporter, 0.5 ng/well of pRL-SV40 normalisation plasmid and 150 ng/well of the corresponding expression plasmid as described above. All experiments were performed in triplicates.

### Animal Experiments

Male 8-week-old C57Bl6 mice received either a single or daily intraperitoneal injections of dexamethasone (1 mg/kg body weight) or saline for 23 days and were analyzed 3 hours after the final DEX injection. After sacrifice, the liver was collected, snap-frozen and used for further analysis. Total RNA was extracted from homogenized liver using the RNeasy Kit (Qiagen). Animal handling and experimentation of this study were done in accordance with NIH guidelines and approved by the german animal welfare authorities (Regierungspräsidium Karlsruhe, permit number 35–9185.81/G-90/11).

### RT-PCR

MCF-10A cells were subjected to 96 h of steroid hormone withdrawal, followed by drug treatment for the indicated time as described above. Then cells were lysed as described previously [33] and RNA was isolated using the RNeasy Kit (Qiagen). cDNA was obtained from reverse transcription of 1 µg of total RNA using 10 ng/µl of p(dN)_6_ random primers (Roche) and SuperScript II reverse transcriptase (Invitrogen) in a final volume of 20 µl according to the manufacturer’s instructions. 1 µl of cDNA was subsequently used for semi-quantitative as well as for quantitative real time PCRs. Semi-quantitative PCRs were carried out using Platinum Taq DNA polymerase (Invitrogen) and were monitored within the linear range of the reaction on a 1% (wt/vol) agarose gel followed by ethidium bromide staining. PCR conditions for each reaction, including primers, annealing temperature, amplicon size and number of cycles, are listed in table S1. Quantitative real time PCRs were performed using LightCycler FastStart DNA Master^PLUS^ SYBR Green I (Roche) according to the manufacturer’s instructions in a Roche LightCycler 1.5 instrument (Roche) with the following amplification parameters: initial denaturation at 95°C for 60 s, followed by 50 cycles of 95°C for 10 s, annealing temperature as indicated in table S1 for 5 s and elongation at 72°C for 20 s. The temperature transition rate was 20°C/s. Primers used are listed in table S1. The specificity of each primer was confirmed by melting curve analysis. Real-time data was analysed using the LightCycler software version 3.5 (Roche). Glyceraldehyde 3-phosphate dehydrogenase (GAPDH) was amplified as an internal control. The samples were loaded in triplicate, and the results of each sample were normalized to GAPDH to obtain an average ± standard deviation (SD).

### Western Blotting

For western blot analysis, total protein content was determined according to Bradford. Cell lysates were separated by SDS-polyacrylamide gel electrophoresis (10 µg total protein per lane) on a 10% gel and transferred onto a PVDF-membrane (Millipore) using a TE 77 semi-dry transfer unit (Amersham Bioscience). Membranes were then blocked overnight at 4°C, using 5% of milk powder in TBST (0.15 M NaCl, 10 mM Tris, 0.05% (v/v) Tween 20, pH 8.0). Proteins of interest were visualized using the antibodies described above and the enhanced chemoluminescence substrate Plus-ECL (PerkinElmer). To measure phospho-FOXO3/FOXO3 ratios, films were scanned and quantified using the ImageJ software (version 1.45s, National Institutes of Health, USA).

### Chromatin Immunoprecipitation (ChIP)

ChIP assays were performed as described previously [33]. Briefly, MCF-10A cells were starved of steroids for 96 h in DMEM/F12 supplemented with 2% charcoal stripped FBS and then treated with 1 µM dexamethasone or vehicle (0.1% ethanol) for 3 h. Cells were fixed with 1% formaldehyde, lysed and sonicated. Lysates were incubated with either a GR-specific antibody or an unspecific control IgG. Recovered DNA was analysed by PCR using the following primers: ChIP A forward, 5′-CAGCTCATAGCTGTTTGGTACACGGTGTTCAA-3′; ChIP A reverse, 5′-AGAGCCCACATTCTAGGAACTGAGCCTACTGC-3′ (position: -4253 to - 3988; length: 265 bp); ChIP B forward, 5′-AGCCACCGCAGTAAGCAGTCAGAGCCC-3′; ChIP B reverse, 5′-CAAAGCCTCCTGTTTGTGCTTCGCCCA-3′ (position: -2440 to -2149; length: 297 bp); ChIP C forward 5′-GCGAGCTGACAGGCGGTTCC-3′; ChIP C reverse 5′- CGCTGCTGCCATCTTGACAGTTTCC-3′ (position: +44 to +370; length: 326 bp). All PCR products were analysed within the linear range of the reaction on a 1% (wt/vol) agarose gel followed by ethidium bromide staining.

### Electrophoretic Mobility Shift Assay (EMSA)

200 ng of annealed synthetic oligonucleotide probes (Table 1) were end-labeled with 6000 Ci/mmol [γ-^32^P] ATP (Perkin Elmer, Boston, USA) by 10 U of T4 polynucleotide kinase (New England Biolabs) in a final reaction volume of 10 µl for 30 min at 37°C and purified from a 15% non-denaturing polyacrylamide gel. Approximately 0.2 ng (10–15000 cpm) of the probe was incubated together with either 25 ng of full length GST-tagged FOXO3 protein (Abnova, Taipei, Taiwan) or 2–4 µg of nuclear cell extracts from MCF-10A cells for 30 min at 25°C in a final volume of 20 µl binding buffer containing 10% glycerol, 12 mM HEPES, pH 7.9, 4 mM Tris-HCl, pH 7.9, 60 mM KCl, 1 mM dithiothreitol, 0.6 mg/ml bovine serum albumin, 0.5 µg of poly(dI-dC) (Sigma) and competitor as indicated. For the antibody incubation assay, 2 µg of a FOXO3 specific antibody was added. Subsequently the binding reaction was separated on a 4% polyacrylamide gel in 1X TB 90 mM Tris, 90 mM boric acid).

### Statistical Analysis

A one-way analysis of variance (ANOVA) using the GraphPad PRISM® program Version 5.0 followed by a Newman-Keuls post-hoc test was performed for statistical analysis of the results shown in Figure 5, 6 and 8A, and a two-way ANOVA followed by Bonferroni analysis was performed for statistical analysis of the results shown in Figure 1 and 8B. A t-test was performed for statistical analysis of the results shown in Figure 3 and 4. Differences with a p value <0.05 were considered statistically significant. One * symbol indicates differences with a p value <0.05, two ** and three *** symbols indicate differences with p values <0.01 or <0.001, respectively.

## Supporting Information

Figure S1 **Anti-phospho-FOXO3 (S413) antibody recognizes FOXO3 only when phosphorylated at the AMPK phosphorylation site S413.** HEK 293 cells transfected with either an empty vector control (pcDNA3), a FOXO3 wild-type expression plasmid or a FOXO3 expression plasmid in which Ser 413 was mutated to Ala, were treated with or without 50 mM 2-Deoxyglucose (2-DG) to activate AMPK. Lysates (10 µg of total protein) were treated with or without lambda phosphatase (λ-Phos) and separated on a 10% (wt/vol) polyacrylamide gel. Relative protein levels of phospho-FOXO3 (S413), total FOXO3 and actin as internal control were analysed by western blotting. The ratios of p-FOXO3/FOXO3 for each experimental condition are indicated. Either lambda phosphatase treatment or mutation of Ser 413 to Ala completely inhibited the detection of phospho-FOXO3 (S413), confirming that the anti-phospho-FOXO3 (S413) antibody recognizes FOXO3 only when phosphorylated at S413.
(TIF)

Figure S2 SGK-1 mediated **inhibitory FOXO3 phosphorylation at T32 is abrogated by concomitant treatment with AMPK-activating 2-Deoxyglucose.** MCF-10A cells were treated with 50 mM 2-Deoxyglucose (to activate AMPK) in combination with either vehicle (Ctrl), 1 µM dexamethasone (DEX) or a mixture of 1 µM dexamethasone and 1 µM RU-486 (DEX/RU) for 18 h. Relative protein levels of phospho-AMPK (T172), phospho-FOXO3 (T32), total FOXO3 and actin as internal control were analysed by western blotting. The ratios of p-FOXO3/FOXO3 for each experimental condition are indicated.
(TIF)

Figure S3 **Competition of FOXO3 binding to the E2-site of its own promoter by different FOXO3 binding sites confirms high affinity binding to FOXO3 promoter.** Complex formation between GST-FOXO3 and the ^32^P-labeled wild-type FOXO3 E2 binding site of the FOXO3 promoter (FOXO3 E2 Wt) was competed with increasing concentrations of unlabeled oligonucleotides (0–200 nM) containing either the FOXO3 site of the LKB1 promoter (LKB1 FOXO, lanes 3–7) or the wild-type FOXO3 E2-site (FOXO3 E2 Wt, lanes 8–12). Addition of a GST specific antibody (α-GST, comparison lanes 1 and 2) confirmed that the composition of protein-DNA complexes consists of FOXO3 molecules.
(TIF)

Table S1 **Primer list and RT-PCR conditions.** Sequences of primers used for semi-quantitative and quantitative RT-PCRs. The annealing temperature, the amplicon size as well as the number of conducted cycles is indicated.
(DOC)
