# Supplementary figures and images for: FOXO3 Is a Glucocorticoid Receptor Target and Regulates LKB1 and Its Own Expression Based on Cellular AMP Levels via a Positive Autoregulatory Loop
Source: PLoS One. 2012 Jul 27;7(7):e42166. doi: 10.1371/journal.pone.0042166 (PMC3407083; doi:10.1371/journal.pone.0042166)

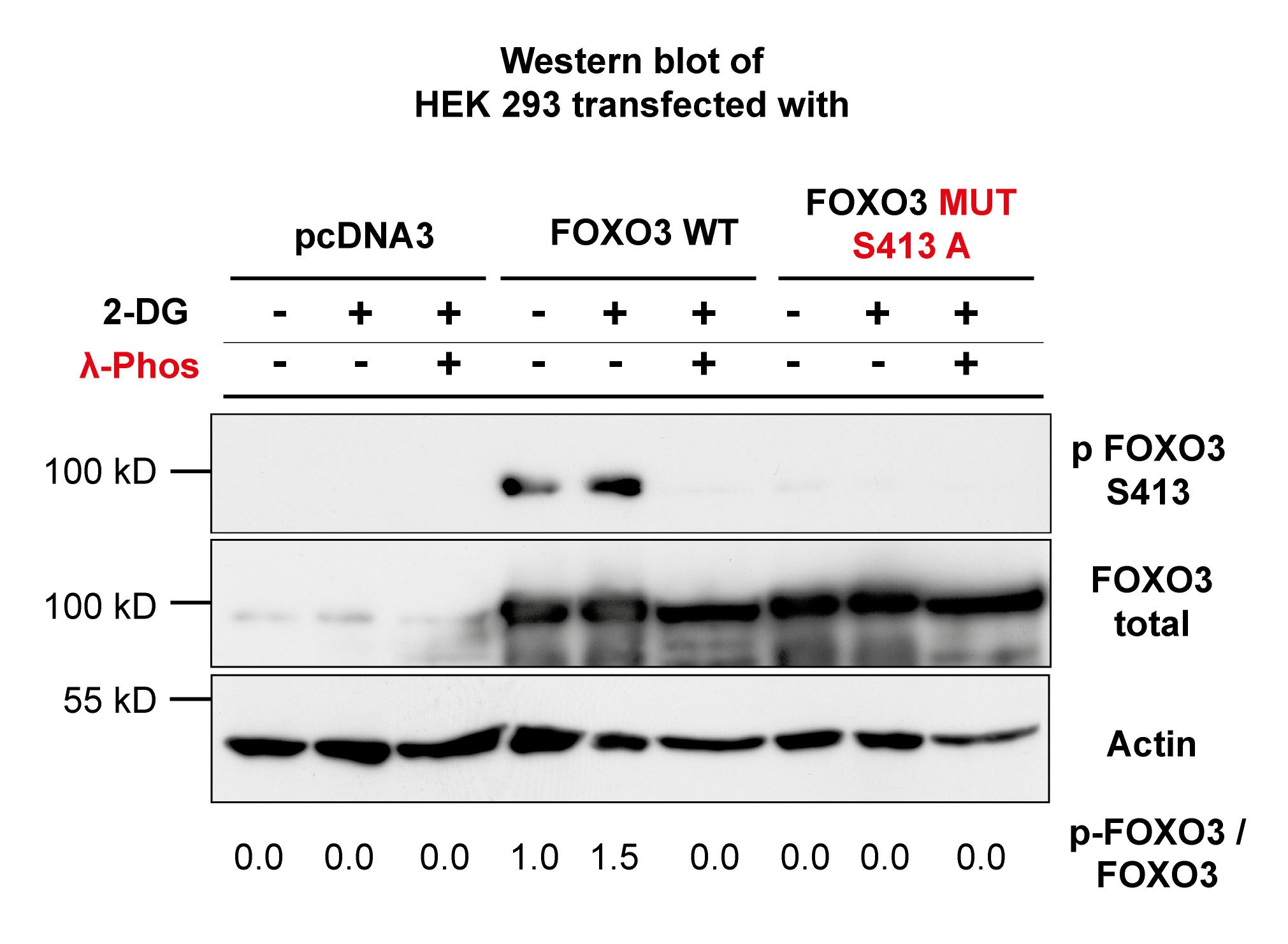

Supplement: Figure S1 — Anti-phospho-FOXO3 (S413) antibody recognizes FOXO3 only when phosphorylated at the AMPK phosphorylation site S413. HEK 293 cells transfected with either an empty vector control (pcDNA3), a FOXO3 wild-type expression plasmid or a FOXO3 expression plasmid in which Ser 413 was mutated to Ala, were treated with or without 50 mM 2-Deoxyglucose (2-DG) to activate AMPK. Lysates (10 µg of total protein) were treated with or without lambda phosphatase (λ-Phos) and separated on a 10% (wt/vol) polyacrylamide gel. Relative protein levels of phospho-FOXO3 (S413), total FOXO3 and actin as internal control were analysed by western blotting. The ratios of p-FOXO3/FOXO3 for each experimental condition are indicated. Either lambda phosphatase treatment or mutation of Ser 413 to Ala completely inhibited the detection of phospho-FOXO3 (S413), confirming that the anti-phospho-FOXO3 (S413) antibody recognizes FOXO3 only when phosphorylated at S413. (TIF) [file pone.0042166.s001.tif]

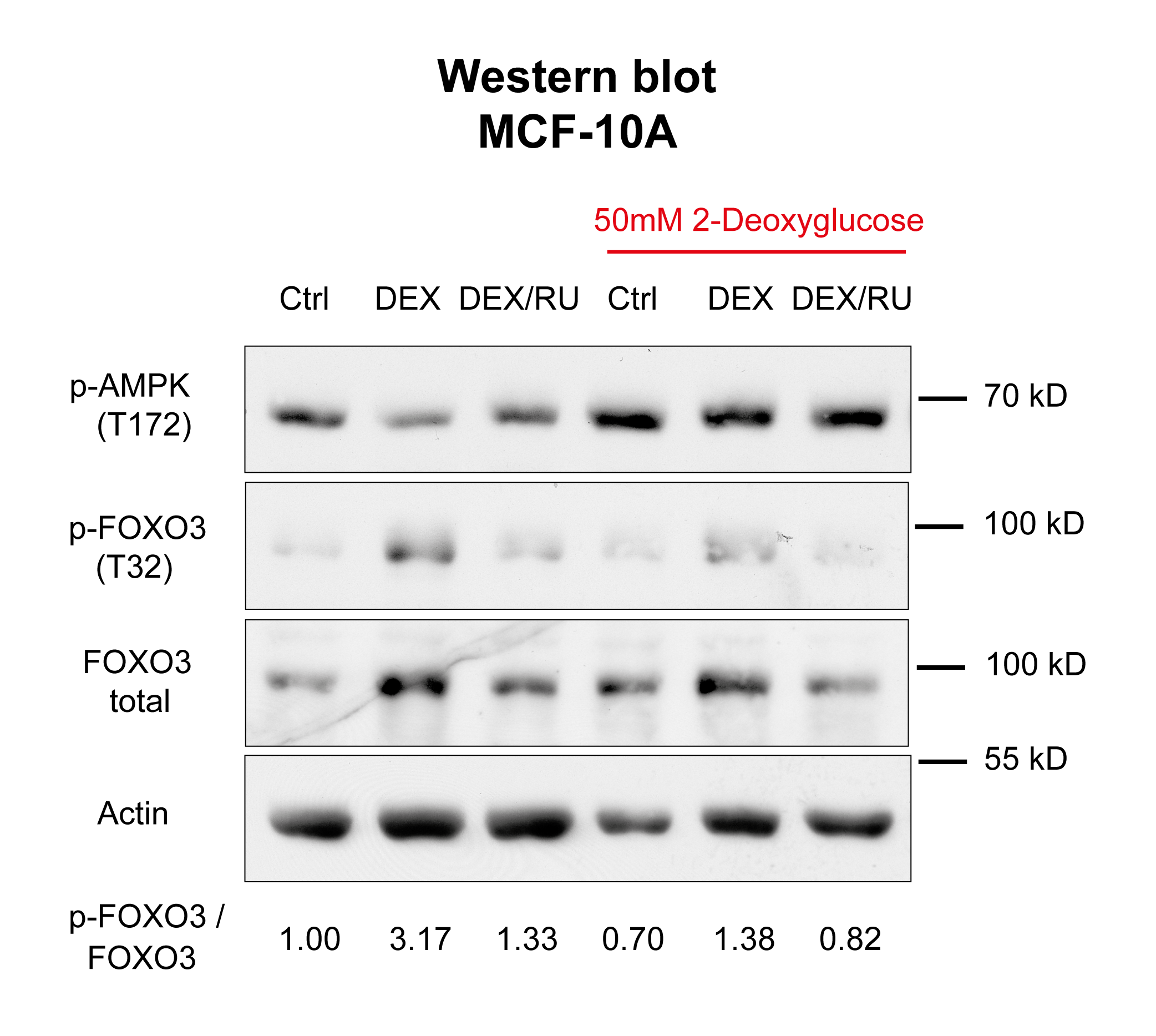

Supplement: Figure S2 — SGK-1 mediated inhibitory FOXO3 phosphorylation at T32 is abrogated by concomitant treatment with AMPK-activating 2-Deoxyglucose. MCF-10A cells were treated with 50 mM 2-Deoxyglucose (to activate AMPK) in combination with either vehicle (Ctrl), 1 µM dexamethasone (DEX) or a mixture of 1 µM dexamethasone and 1 µM RU-486 (DEX/RU) for 18 h. Relative protein levels of phospho-AMPK (T172), phospho-FOXO3 (T32), total FOXO3 and actin as internal control were analysed by western blotting. The ratios of p-FOXO3/FOXO3 for each experimental condition are indicated. (TIF) [file pone.0042166.s002.tif]

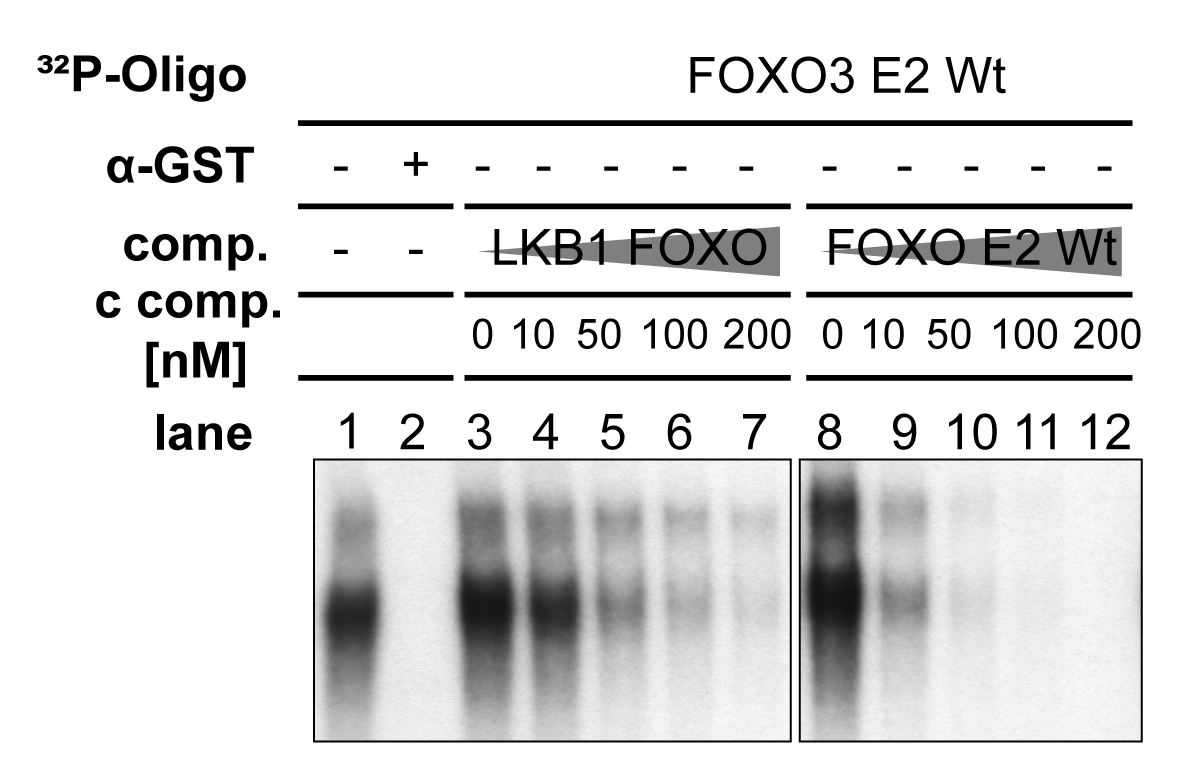

Supplement: Figure S3 — Competition of FOXO3 binding to the E2-site of its own promoter by different FOXO3 binding sites confirms high affinity binding to FOXO3 promoter. Complex formation between GST-FOXO3 and the 32P-labeled wild-type FOXO3 E2 binding site of the FOXO3 promoter (FOXO3 E2 Wt) was competed with increasing concentrations of unlabeled oligonucleotides (0–200 nM) containing either the FOXO3 site of the LKB1 promoter (LKB1 FOXO, lanes 3–7) or the wild-type FOXO3 E2-site (FOXO3 E2 Wt, lanes 8–12). Addition of a GST specific antibody (α-GST, comparison lanes 1 and 2) confirmed that the composition of protein-DNA complexes consists of FOXO3 molecules. (TIF) [file pone.0042166.s003.tif]
