# Supplementary material for: FOXO3 Is a Glucocorticoid Receptor Target and Regulates LKB1 and Its Own Expression Based on Cellular AMP Levels via a Positive Autoregulatory Loop
Source: PLoS One. 2012 Jul 27;7(7):e42166. doi: 10.1371/journal.pone.0042166 (PMC3407083; doi:10.1371/journal.pone.0042166)
Supplement: Table S1 — Primer list and RT-PCR conditions. Sequences of primers used for semi-quantitative and quantitative RT-PCRs. The annealing temperature, the amplicon size as well as the number of conducted cycles is indicated. (DOC) [file pone.0042166.s004.doc]

Table S1. Primer list and RT-PCR conditions.

| Semi-Quantitative RT-PCRs | | | | |
| --- | --- | --- | --- | --- |
|  | | | | |
| Human Amplicons | | | | |
| **Primer name** | **Sequence** | Annealing  temperature | Amplicon  size | Conducted  Cycles  (Within linear range) |
| FOXO3 FW | GGGAAACCTGTCCTACGCGG | 57.5°C | 407 bp | 27 |
| FOXO3 RV | CAGGCCACTTGGAGAGCTGG |
| GAPDH FW | GCCACATCGCTCAGACACCA | 59°C | 291 bp | 22 |
| GAPDH RV | CCAGCATCGCCCCACTTGAT |
| IGFBP3 FW | CAGAGACTCGAGCACAGCAC | 55°C | 192 bp | 27 |
| IGFBP3 RV | ATGACCGGGGTTTAAAGGT |
| LKB1 FW | GAGCTGATGTCGGTGGGTAT | 55°C | 269 bp | 30 |
| LKB1 RV | CTTCACCTTGCCGTAAGAGC |
| NF-YB FW | GGTGCCATCAAGAGAAACGG | 55.5°C | 161 bp | 29 |
| NF-YB RV | AGCTGTGACTGCTCCACCAA |
| SGK-1 FW | GGAGCCTGAGCTTATGAATGCCAAC | 59°C | 586 bp | 26 |
| SGK-1 RV | TGCCACAGAAGGTGGATGTTGTGC |
| Sp1 FW | GCCGCTCCCAACTTACAGAACCAGC | 58°C | 399 bp | 25 |
| Sp1 RV | CCTTGACTCCCAGCTCTCAGGCAG |
| β-Tubulin FW | tctgttcgctcaggtccttt | 59°C | 271 bp | 25 |
| β-Tubulin RW | Ttcatgatgcgatcagggta |
| TRAIL FW | Ccagaggaagaagcaacaca | 55°C | 109 bp | 30 |
| TRAIL RW | GGAATGAATGCCCACTCC |
|  |  |  |  |  |
| Mouse Amplicons | | | | |
| mFoxo3 FW | GCTCCCCGGACAAACGGCTC | 59°C | 362 bp | 27 |
| mFoxo3 RV | CAGGCCACTTGGAGAGCTGG |
| mGAPDH FW | CTTCATTGACCTCAACTACATGGTC | 58°C | 440 bp | 20 |
| mGAPDH RV | GCAGTGATGGCATGGACTGTG |
|  |  |  |  |  |
|  |  |  |  |  |
|  |  |  |  |  |
| Quantitative RT-PCRs | | | | |
|  | | | | |
| Human Amplicons | | | | |
| FOXO3 FW | GGGAAACCTGTCCTACGCGG | 57°C | 407 bp |  |
| FOXO3 RV | CAGGCCACTTGGAGAGCTGG |
| GAPDH FW | GCCACATCGCTCAGACACCA | 57°C | 291 bp |  |
| GAPDH RV | CCAGCATCGCCCCACTTGAT |
| LKB1 FW | GAGCTGATGTCGGTGGGTAT | 54°C | 269 bp |  |
| LKB1 RV | CTTCACCTTGCCGTAAGAGC |
|  |  |  |  |  |
| Mouse Amplicons | | | | |
| mFoxo3Q FW | GGATGACGTCCAGGATGATG | 57°C | 237 bp |  |
| mFoxo3Q RV | GAGCCCCGCTGCATAAGCCC |
| mGAPDH FW | CTTCATTGACCTCAACTACATGGTC | 57°C | 440 bp |  |
| mGAPDH RV | GCAGTGATGGCATGGACTGTG |
